# Identification and Prediction of Clinical Phenotypes in Hospitalized Patients With COVID-19: Machine Learning From Medical Records

**DOI:** 10.2196/46807

**Published:** 2023-10-06

**Authors:** Tom Velez, Tony Wang, Brian Garibaldi, Eric Singman, Ioannis Koutroulis

**Affiliations:** 1 Computer Technology Associates Cardiff, CA United States; 2 Imedacs Ann Arbor, MI United States; 3 Biocontainment Unit Division of Pulmonary and Critical Care Medicine Johns Hopkins University School of Medicine Baltimore, MD United States; 4 Department of Ophthalmology and Visual Sciences University of Maryland School of Medicine Baltimore, MD United States; 5 Department of Neurology University of Maryland School of Medicine Baltimore, MD United States; 6 Division of Emergency Medicine Childrens National Hospital Washington, DC United States

**Keywords:** big data, COVID, respiratory distress, critical care, early warning, electronic medical record, machine learning, clinical phenotypes, pathogenesis, infection, immune response, treatment, biomarkers, training, sepsis, mortality, utility, phenotype, support tool

## Abstract

**Background:**

There is significant heterogeneity in disease progression among hospitalized patients with COVID-19. The pathogenesis of SARS-CoV-2 infection is attributed to a complex interplay between virus and host immune response that in some patients unpredictably and rapidly leads to “hyperinflammation” associated with increased risk of mortality. The early identification of patients at risk of progression to hyperinflammation may help inform timely therapeutic decisions and lead to improved outcomes.

**Objective:**

The primary objective of this study was to use machine learning to reproducibly identify specific risk-stratifying clinical phenotypes across hospitalized patients with COVID-19 and compare treatment response characteristics and outcomes. A secondary objective was to derive a predictive phenotype classification model using routinely available early encounter data that may be useful in informing optimal COVID-19 bedside clinical management.

**Methods:**

This was a retrospective analysis of electronic health record data of adult patients (N=4379) who were admitted to a Johns Hopkins Health System hospital for COVID-19 treatment from 2020 to 2021. Phenotypes were identified by clustering 38 routine clinical observations recorded during inpatient care. To examine the reproducibility and validity of the derived phenotypes, patient data were randomly divided into 2 cohorts, and clustering analysis was performed independently for each cohort. A predictive phenotype classifier using the gradient-boosting machine method was derived using routine clinical observations recorded during the first 6 hours following admission.

**Results:**

A total of 2 phenotypes (designated as phenotype 1 and phenotype 2) were identified in patients admitted for COVID-19 in both the training and validation cohorts with similar distributions of features, correlations with biomarkers, treatments, comorbidities, and outcomes. In both the training and validation cohorts, phenotype-2 patients were older; had elevated markers of inflammation; and were at an increased risk of requiring intensive care unit–level care, developing sepsis, and mortality compared with phenotype-1 patients. The gradient-boosting machine phenotype prediction model yielded an area under the curve of 0.89 and a positive predictive value of 0.83.

**Conclusions:**

Using machine learning clustering, we identified and internally validated 2 clinical COVID-19 phenotypes with distinct treatment or response characteristics consistent with similar 2-phenotype models derived from other hospitalized populations with COVID-19, supporting the reliability and generalizability of these findings. COVID-19 phenotypes can be accurately identified using machine learning models based on readily available early encounter clinical data. A phenotype prediction model based on early encounter data may be clinically useful for timely bedside risk stratification and treatment personalization.

## Introduction

### Background

Among hospitalized patients with COVID-19, there is significant interindividual variability. A significant number (20%-67%) progress from moderate illness to life-threatening complications, including acute respiratory distress syndrome (ARDS) [1,2] and septic shock [3], generating a surge in patients who require intensive care unit (ICU)–level respiratory and vasopressor support [4]. Among patients with COVID-19 who are critically ill and require invasive mechanical ventilation, a delay in intubation from the first noninvasive respiratory support is associated with an increase in hospital mortality [4]. Similarly, delayed vasopressor initiation in patients with septic shock has been found to be associated with increased mortality [5]. Acute kidney injury (AKI) is also common among hospitalized patients with COVID-19 and is associated with high mortality [6]. In a recent observational study of 3993 hospitalized patients with COVID-19, AKI occurred in 46% of patients, and 19% required dialysis [7]. A recent meta-analysis of 34 observational studies of hospitalized patients found that delayed ICU admission was remarkably associated with mortality, highlighting the importance of providing timely critical care in non-ICU settings [8].

To support risk stratification among heterogeneous hospitalized patients, recent studies have used machine learning–based clustering [9] to retrospectively analyze routinely available patient electronic health record (EHR) data to identify clinically useful phenotypes [10]. In critical care research, unsupervised machine learning clustering has been used to identify homogeneous subgroups within a broad heterogeneous hospitalized population [11], which elucidates pathophysiology, can predict treatment response, and has the potential to augment clinical trial enrollment [10]. The most common clustering techniques used in medicine are latent class analysis (LCA), an algorithm that derives clusters using a probabilistic model that describes the distribution of the data [12,13], and k-means, which identifies clusters in a data set by using a distance metric to find k centroids (a weighted average) within the n-dimensional space of clinical features [11-14]. Both LCA and k-means have been effectively [15] used to detect homogeneous phenotypes with distinct severities and treatment responses in ARDS [16-18], sepsis [19,20], and COVID-19 [21,22].

In support of point-of-care clinical management, modern predictive machine learning classification algorithms (eg, the gradient-boosting machine [GBM] algorithm [23]) trained using features based on observations recorded early in a new encounter have shown promise in rapidly assigning de novo patients to a clustering-identified phenotype [24]. GBM classifiers are increasingly being applied for prediction in the data science industry and are known to outperform simpler models such as logistic regression in many clinical research fields, including critical care [25,26]. GBM has been used to accurately identify LCA-derived ARDS phenotypes [24], including a hyperinflammatory phenotype characterized by elevated inflammatory biomarkers, higher prevalence of vasopressor use, longer use of ventilation, extended length of stay, higher prevalence of sepsis, and higher mortality [27-31]. In addition, a recent ARDS study observed differential responses to positive end-expiratory pressure strategy by phenotype, with higher positive end-expiratory pressure associated with improved outcomes in the hyperinflammatory phenotype [27].

A recently reported EHR data clustering analysis of a relatively small sample of patients with COVID-19 admitted to a US hospital identified 2 phenotypes designated as cluster 1 and cluster 2 [21]. Patients in cluster 1 were older individuals (mean age 79.5 years) with multiple comorbidities and a higher mortality rate (25.4% vs 8.97%; *P*<.001) than patients in cluster 2. Patients in cluster 2 were younger individuals (mean age 53.7 years) who were more likely to be male and racial and ethnic minority individuals with higher levels of inflammatory markers and alanine aminotransferase (ALT) and a markedly increased BMI.

### Objectives

In this study, we sought to explore the generalizability of this 2-phenotype finding for COVID-19 using a clustering analysis of EHR data associated with a much larger cohort of hospitalized patients. Analogous to the ARDS study cited previously, we also explored the application of GBM-based phenotype classifier algorithms trained using routinely available clinical data for the rapid identification of clustering-derived COVID-19 phenotypes.

## Methods

### Overview

Deidentified EHR data were extracted from the JH-CROWN Registry [32] on patients with COVID-19 who were admitted to the Johns Hopkins (JH) Health System from February 25, 2020, to March 3, 2021. The registry, constructed directly from the JH clinical EHR, was designed to serve as a comprehensive projection of structured clinical data for patients with COVID-19. Diagnosis of COVID-19 was defined as a positive molecular test for SARS-CoV-2 and either a COVID-19 International Classification of Diseases, 10th Revision, diagnosis or an associated diagnosis suggesting that COVID-19 was likely present (eg, pneumonia, ARDS, or anosmia). Patients transferred from other health care institutions were excluded. Given that our goal was to identify phenotypes potentially at high risk of deterioration, we also excluded patients who had initiated critical care treatment (eg, invasive mechanical ventilation or dialysis) or died during the 6-hour time window following admission. The extracted registry data included patient demographics, encounter information, problem lists, diagnoses, flow sheets, laboratory test results, medications, procedures, and outcomes associated with the patients (N=4379).

### Clustering to Identify COVID-19 Phenotypes

**Figure 1:**
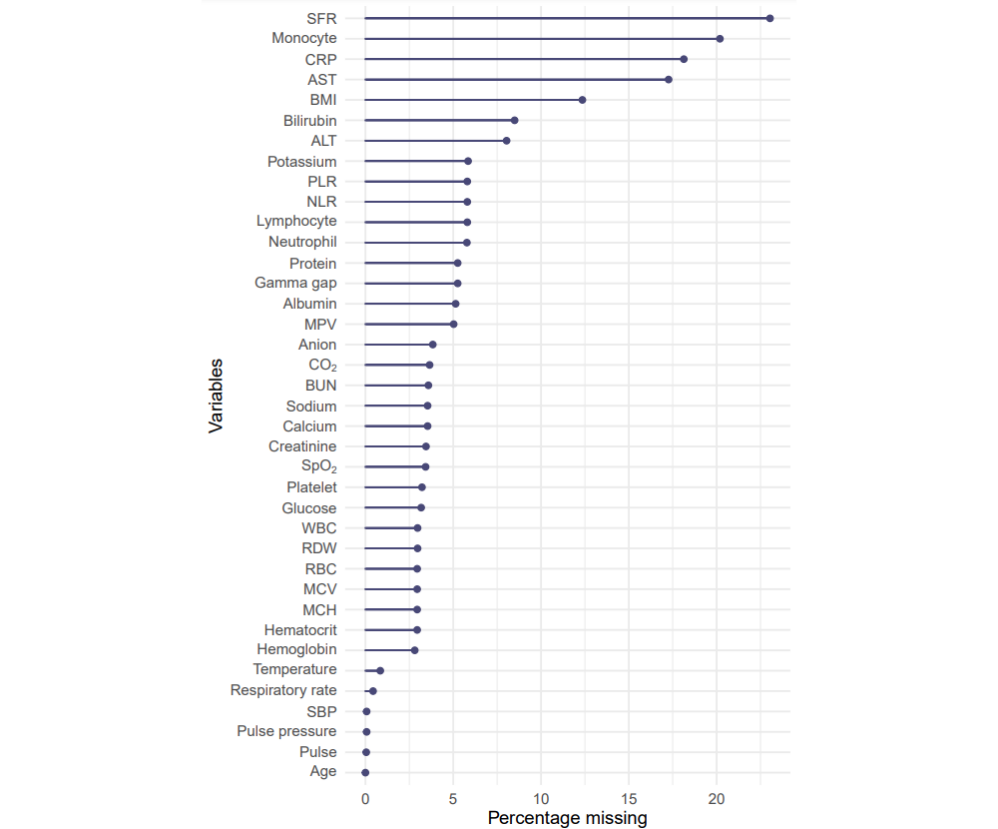
Missingness of clinical observations used for clustering. Clinical physiological observations associated with included patients (adults; nontransferees) with missingness of <25% in the population (N=4379) over the entire encounter. ALT: alanine transaminase; AST: aspartate aminotransferase; BUN: blood urea nitrogen; CO2: carbon dioxide; CRP: C-reactive protein; MCH: mean corpuscular hemoglobin; MCV: mean corpuscular volume; MPV: mean platelet volume; NLR: neutrophil-to-lymphocyte ratio; PLR: platelet-to-lymphocyte ratio; RBC: red blood cell count; RDW: red cell distribution width; SBP: systolic blood pressure; SFR: oxygen saturation–to–fraction of inspired oxygen ratio; SpO2: oxygen saturation; WBC: white blood cell count.

**Figure 2:**
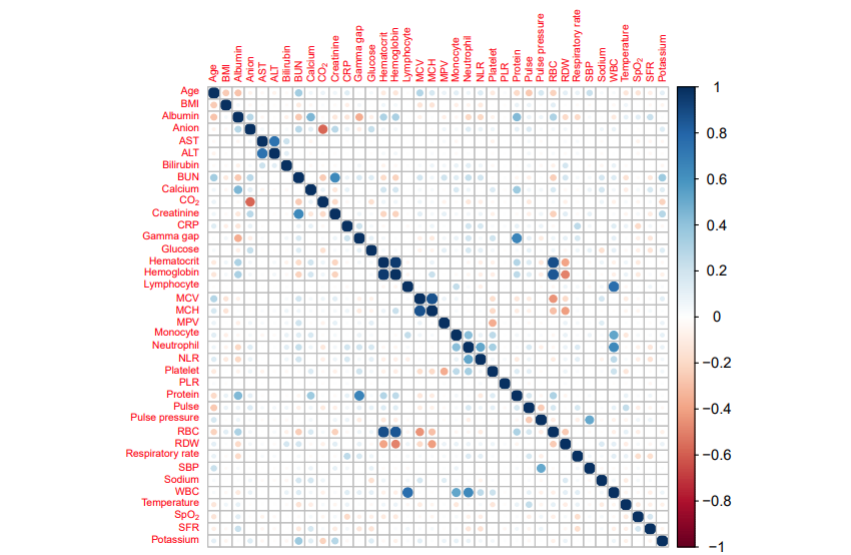
Heat map of correlations among clinical data used to generate clustering features showing highly uncorrelated data except for expected positive correlations between red blood cell count (RBC), hemoglobin, and hematocrit and correlations between white blood cell count (WBC) and lymphocytes or neutrophils. ALT: alanine transaminase; AST: aspartate aminotransferase; BUN: blood urea nitrogen; CO2: carbon dioxide; CRP: C-reactive protein; MCH: mean corpuscular hemoglobin; MCV: mean corpuscular volume; MPV: mean platelet volume; NLR: neutrophil-to-lymphocyte ratio; PLR: platelet-to-lymphocyte ratio; RDW: red cell distribution width; SBP: systolic blood pressure; SFR: oxygen saturation–to–fraction of inspired oxygen ratio; SpO2: oxygen saturation.

Data used for clustering included age, BMI, and 36 clinical observations (vitals and laboratory tests) selected based on registry data availability (<25% missingness, as shown in Figure 1) associated with the included patients with COVID-19. Figure 2 is a correlation heat map showing that our selected data elements were mostly uncorrelated except for expected strong associations in observations, such as between creatinine and blood urea nitrogen [33]; among white blood cell count, lymphocytes, and neutrophils; and among red blood cell count, hemoglobin, and hematocrit [34]. Clustering features were generated as minimums or maximums of these vitals and laboratory tests [31] within the context of severe COVID-19 illness recorded during the entire hospital stay (Textbox 1).

**Clinical features used for clustering.**

**Vitals**

- Minimum: oxygen saturation (SpO_2_), SpO_2_/fraction of inspired oxygen, systolic blood pressure, and pulse pressure
- Maximum: pulse, respiratory rate, and temperature

**Laboratory tests**

- Minimum: albumin, calcium, carbon dioxide, gamma gap, hematocrit, hemoglobin, lymphocytes, mean corpuscular hemoglobin, mean corpuscular volume, monocytes, platelets, protein, potassium, red blood cell count, red cell distribution width, and sodium
- Maximum: aspartate transferase, alanine aminotransferase, anion, bilirubin, blood urea nitrogen, creatinine, C-reactive protein, glucose, mean platelet volume, neutrophils, neutrophil-to-lymphocyte ratio, platelet-to-lymphocyte ratio, and white blood cell count

The practice of splitting a data set into training and validation data sets toward assessments of generalizability of machine learning–based subgroup discovery is well established [35]. Accordingly, in our study, patient encounters were split into 2 cohorts randomly. Cohort 1 (2179/4379, 49.76%) was used as the training cohort, and cohort 2 (2182/4379, 49.83%) served as the internal validation set. Table 1 shows the basic demographics, comorbidities, vitals, and inflammation biomarkers associated with the full cohort as well as the highly similar training and validation cohorts. Following data cleansing to account for data outliers, as described in more detail in the following sections, missing data imputation and clustering analysis for each cohort were independently performed. Beyond comparing overall clustering result indexes such as the number of phenotypes identified and how Textbox 1 features were statistically distributed across the phenotypes identified in the 2 data sets (ie, internal validation [35]), we also explored the similarity of clinical data distributions across phenotypes detected in the training and validation cohorts *not* used for clustering (ie, external validation [35]). The features used for external validation included inflammatory biomarkers not used for clustering because of excessive missingness (eg, D-dimer and ferritin), treatment response (eg, the need for critical care treatment such as invasive mechanical ventilation, dialysis, and vasopressors), or outcomes (eg, length of stay, sepsis, and survival).

**Table 1:** Basic demographics, comorbidities, vitals, and inflammation biomarkers associated with the full study cohort and the split training and validation cohorts used for clustering analysis.

Selected clinical characteristics				Full cohort (N=4379)		Training (n=2182)		Validation (n=2197)
**Basic demographics and comorbidities**								
	Age (years), median (IQR)		62 (48-75)		62 (47-75)		61 (47-75)	
	Sex (male), n (%)		2141 (48.89)		1047 (47.98)		1094 (49.8)	
	**Race and ethnicity, n (%)**							
		Asian	247 (5.64)		140 (6.41)		107 (4.87)	
		Black	1552 (35.44)		786 (36.02)		766 (34.87)	
		Hispanic	840 (19.18)		403 (18.46)		437 (19.89)	
		Non-Hispanic White	1543 (35.23)		757 (34.69)		786 (35.77)	
	Hypertension, n (%)		2867 (65.47)		1436 (65.81)		1431 (65.13)	
	Lymphoma, n (%)		79 (1.8)		43 (1.97)		36 (1.63)	
	Congestive heart failure, n (%)		848 (19.36)		431 (19.75)		417 (18.98)	
	Renal failure, n (%)		1043 (23.81)		517 (23.69)		526 (23.94)	
	Peripheral vascular disease, n (%)		615 (14.04)		289 (13.24)		326 (14.83)	
	AIDS, n (%)		81 (1.85)		41 (1.87)		40 (1.82)	
	Chronic pulmonary disease, n (%)		1220 (27.86)		609 (27.91)		611 (27.81)	
	Metastatic cancer, n (%)		297 (6.78)		160 (7.33)		137 (6.23)	
	Liver disease, n (%)		522 (11.92)		259 (11.86)		263 (11.97)	
	Diabetes with chronic complications, n (%)		1324 (30.23)		632 (28.96)		692 (31.49)	
	Valvular disease, n (%)		478 (10.92)		255 (11.68)		223 (10.15)	
**Vitals and inflammation biomarkers, median (IQR)**								
	BMI (kg/m^2^)^a^		28.3 (24.1-33.8)		28.2 (24.2-33.5)		29.3 (24.1-24.1)	
	Maximum pulse (beats per min)		111.0 (98.0-126.0)		111.0 (98.0-126.0)		111.0 (98.0-126.0)	
	Maximum respiratory rate (breaths per min)		27.0 (22.0-36.0)		28.0 (22.0-36.0)		27.0 (22.0-36.0)	
	Maximum temperature (°F)		100.6 (99.4-102.2)		100.4 (99.4-102.2)		100.6 (99.5-102.2)	
	Minimum SpO_2_^b^ (%)		90.0 (85.0-93.0)		90.0 (85.0-93.0)		90.0 (85.0-93.0)	
	Minimum SpO_2_/FiO_2_^c,d^		438.1 (325.0-476.2)		438.1 (321.4-476.2)		438.1 (325.5-476.2)	
	Minimum systolic BP^e^ (mm Hg)		96.0 (85.0-105.0)		95.0 (85.0-105.0)		96.0 (85.0-106.0)	
	Minimum pulse pressure (mm Hg)		96.0 (85.0-105.0)		95.0 (85.0-105.0)		96.0 (85.0-106.0)	
	Maximum WBC^f^ (K/cu mm)^g^		9.65 (6.8-13.7)		9.71 (6.8-13.7)		9.64 (6.9-13.7)	
	Maximum neutrophils (K/cu mm)^h^		6.92 (4.6-10.7)		6.9 (4.5-10.7)		6.95 (4.7-10.6)	
	Maximum CRP^i^ (mg/dL)^j^		10.8 (4.8-30.0)		11.0 (4.8-31.5)		10.6 (4.8-28.3)	
	Minimum platelets (K/cu mm)^k^		173.0 (133-225)		175 (132-228)		172 (133-222.7)	
	Minimum lymphocytes (K/cu mm)^l^		0.77 (0.48-1.14)		0.77 (0.48-1.14)		0.78 (0.48-1.14)	
	Maximum D-dimer (mg/L)^m^		1.27 (0.67-3.38)		1.27 (0.66-3.51)		1.27 (0.67-3.24)	
	Maximum ferritin (µg/L)^n^		616.5 (283.7-1186.2)		627.5 (283.0-1191.75)		609.5 (286.0-1173.2)	
	Maximum fibrinogen (mg/dL)^o^		506.0 (409.0-633.0)		496.0 (399.0-633.0)		508.0 (407.0-638.0)	
	Maximum IL6^p^ (pg/mL)^q^		34.7 (14.0-77.9)		34.3 (13.8-79.15)		35.7 (14.3-76.8)	
	Maximum LDH^r^ (U/L)^s^		335 (249-479)		334.5 (251-468)		335 (245.5-489.0)	
	Maximum PCT^t^ (ng/mL)^u^		0.25 (0.15-0.65)		0.25 (0.15-0.63)		0.25 (0.15-0.67)	

^a^12.35% (541/4379) of patients with missing data.

^b^SpO_2_: oxygen saturation.

^c^FiO_2_: fraction of inspired oxygen.

^d^23.04% (1009/4379) of patients with missing data.

^e^BP: blood pressure.

^f^WBC: white blood cell count.

^g^0.11% (5/4379) of patients with missing data.

^h^1.32% (58/4379) of patients with missing data.

^i^CRP: C-reactive protein.

^j^14.32% (627/4379) of patients with missing data.

^k^0.11% (5/4379) of patients with missing data.

^l^1.32% (58/4379) of patients with missing data.

^m^11.1% (486/4379) of patients with missing data.

^n^22.63% (991/4379) of patients with missing data.

^o^71.2% (3118/4379) of patients with missing data.

^p^IL6: interleukin 6.

^q^63.14% (2765/4379) of patients with missing data.

^r^LDH: lactate dehydrogenase.

^s^38.05% (1666/4379) of patients with missing data.

^t^PCT: procalcitonin.

^u^61.86% (2709/4379) of patients with missing data.

### Confounding Treatment Bias

A recognized challenge in the use of observational clinical data in machine learning analytics is the need to account for potential biases resulting from treatment that can influence patient physiological measurements [36]. For example, in our study, a significant number of inpatients received supplemental oxygen for acute COVID-19 respiratory symptoms in an emergency room setting before admission, thus potentially biasing observations such as oxygen saturation (SpO_2_) measured following admission. Another potential source of bias can be treatment for hypotension upon presentation, which is known to occur in patients with chronic hypertension [37], triggering the need for fluid boluses or vasopressors before admission. Fortunately, the JH data recorded the start and end times of critical care therapies (eg, high-flow nasal cannula [HFNC], oxygen flow rate [L/min], mechanical ventilation, fraction of inspired oxygen (FiO_2_; %), vasopressors, and dialysis), preadmission treatment, and vital sign information, enabling us to identify the minimums for SpO_2_ and systolic blood pressure before treatment. The potential for SpO_2_ bias owing to supplemental oxygen was also mitigated by the derived ratio of SpO_2_ to FiO_2_. This ratio was calculated using either an FiO_2_ value recorded contemporaneously with SpO_2_ (including cases in which FiO_2_ was recorded as 21%, suggesting that SpO_2_ was a “room air” measurement) or in cases in which an oxygen flow rate was documented (eg, in cases using nasal cannulas) using an estimated FiO_2_ calculated from the oxygen flow rate [38].

### Outliers

Our study explored the detection of phenotypes by clustering routinely available clinical data. However, raw clinical data typically extracted automatically from EHRs can often contain outliers, particularly those associated with observations that may have been manually entered erroneously [20]. Recent studies have confirmed that outliers will negatively affect the quality of derived clusters [39]. Although the JH-CROWN Registry contained syntax error–free structured tables for vitals and laboratory test measurements, unlikely outliers, as recorded in the EHR, were replicated in the registry tables. To cleanse vitals, we adopted reported rules reflecting commonly accepted ranges [40] for human physiology in which outliers were replaced with “null” (ie, treated as “missing”). Table 2 shows the raw total counts of key vitals—with SpO_2_, pulse, and respiration having the highest raw counts followed by temperature and blood pressure—and the statistics of the validated (cleansed) vital signs. For laboratory tests, values were replaced with nulls using the statistical “3(IQR)” criteria designed to detect extreme outliers in observational data [41].

**Table 2:** Statistics on vital signs within acceptable ranges.

	Pulse (beats/min)	Respiratory rate (breaths/min)	Systolic blood pressure (mm Hg)	Diastolic blood pressure (mm Hg)	Temperature (°F)	SpO_2_^a^ (%)
Total count	839,771	704,180	513,758	513,758	520,819	841,647
Acceptable range	30-250	6-60	30-305	20-180	85.1-106.7	60-100
Validated count	839,344	698,933	513,745	504,247	520,602	840,608
Values, mean (SD; range)	86.9 (19.5; 30-247)	22.5 (7.4; 6-60)	125.6 (23.3; 33-272)	69.0 (13.4; 20-166)	98.5 (1.4; 85.3-106.7)	95.8 (3.8; 60-100)

^a^SpO_2_: oxygen saturation.

### Multiple Imputation and Weighted Consensus Clustering

Multiple imputation (MI) and weighted clustering analysis were applied to the training and validation cohorts independently. No validation data were used to influence the imputation of the training data. MI, known to reduce bias even when the proportion of missingness is large [42], is an approach to missing data whereby multiple copies of the feature data set are generated with missing values replaced by inferences drawn from the data set. Our approach was based on Bayesian joint models congenial or compatible with k-means clustering [43,44]. The joint-modeling MI was based on the Dirichlet process mixture of multivariate normal distributions to reflect complex distributional features [45]. For each cohort, a total of 100 imputed data sets were created. K-means clustering was then applied to each imputed data set, generating base clusterings for final weighted consensus clustering.

Although an old rule of thumb is that 3 to 10 imputations would typically suffice to ensure precision and replicability [46], recent studies [47] have developed a new formula based on the fraction of missing information (FMI) that estimates how many imputations would be needed for precise and replicable SE, CIs, *t* statistics, and *P* values [47]. Although, to achieve a variation of <5% in SE at an FMI of 25%, the estimated number of imputations needed is approximately 20 [48], as the FMI increases, the required number of imputations increases quadratically, and at an FMI of 70%, the estimated number of required imputations is approximately 100 [47]. As, in general, adding more imputations increases precision and replicability, we chose 100 imputations to ensure robustness and accuracy, especially in the context of complex data such as the medical records of patients with COVID-19 with a substantial amount of missing data [49].

Although there are numerous methods that have been proposed to determine the optimal number of partitions or clusters in k-means analysis, clustering stability has emerged as a general model-agnostic evaluation method. In statistical learning terms, if data sets are repeatedly and randomly sampled from the same underlying distribution, a stable clustering algorithm should find similar partitions [50]. The approach used in our study defines a “good clustering” in terms of its instability in response to imputation-related perturbations in the data. Instability was assessed using the bootstrapping method [51]. Accordingly, we selected k as the value that minimizes the instability of the clustering [52]. Instability-based methods are attractive as they are not based on a specific metric for the distance between objects and have been shown to perform at least as well as state-of-the-art distance-based methods [53].

Consensus clustering has the theoretical advantage of minimizing overfitting and optimizing the stability of cluster assignments, as has been shown for identifying subgroups of heterogeneous patients in the ICU [10]. A weighted consensus clustering based on the nonnegative matrix factorization (NMF) framework [54] was obtained for the clustering results from all imputed data sets [55]. Unlike a consensus approach based on an averaging process (wherein all base clusterings are treated with equal weight), the objective of NMF weighted consensus clustering is to aggregate the base clusterings into a final clustering using weights optimized for each base clustering in a manner analogous to least absolute shrinkage and selection operator regression [56]. Under this approach, the solution for the weights is sparse (ie, only a small subset of base clusterings contributes to the final clustering). A wide range of comparative experiments has demonstrated the effectiveness of the NMF-based consensus clustering approach [54,55]. The R package *clusterMI* (version 0.0.41; R Foundation for Statistical Computing) [43] was used to perform clustering with MI. In addition, it allows for the consensus pooling of results in terms of both partitions and instability [57].

### Reproducibility

**Figure 3:**
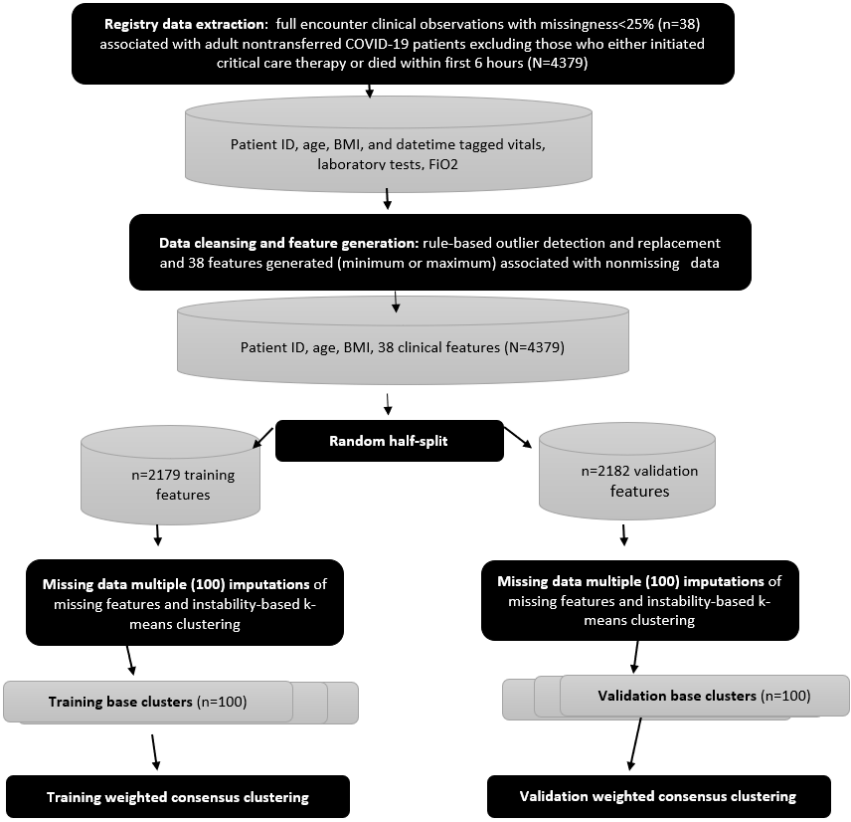
Weighted consensus clustering for COVID-19 phenotype identification process flow. FiO2: fraction of inspired oxygen.

To support the analysis of reproducibility, a statistical analysis of how features and outcomes were distributed across phenotypes in both cohorts, with *P* values established using the Kruskal-Wallis rank sum test for continuous variables and the chi-square test for categorical values [58], was performed. An overview of the clustering process flow is shown in Figure 3.

### Predicting De Novo Patient Phenotype

Although the identification of phenotypes via clustering has the potential to inform personalized care [59], the lack of point-of-care testing of key defining inflammatory biomarkers, especially during the early stages of an encounter, limits the clinical utility of phenotypes. A recent related ARDS study explored the application of supervised GBM phenotype classifiers trained using routinely available observational data and clustering-identified labels and achieved a phenotype classifier with an area under the curve (AUC) of 0.95 [24]. Our study extends this modeling effort by deriving a predictive GBM phenotype classifier trained using data observed within the first 6 hours of admission in which, as in the cited ARDS study, model performance was evaluated against the clustering-derived phenotype.

Our approach to phenotype prediction model development adheres to the TRIPOD (Transparent Reporting of a Multivariable Prediction Model for Individual Prognosis or Diagnosis) guidelines [60]. For prediction modeling, routinely available observational data, as shown in Textbox 1 (except for the C-reactive protein [CRP], which was excluded because of >40% missingness), for included patients (N=4379) recorded within 6 hours following admission were used for training. MI based on Bayesian joint models was applied to create 100 complete feature data sets of the remaining 37 features used for phenotype identification. On each imputed data set, 69.99% (3065/4379) of the included patients were randomly selected as the training set, whereas the remaining 30.01% (1314/4379) were reserved as the test data set. The *imputeData()* function from the aforementioned *clusterMI* R package [57] was used to perform MIs of the early feature data before random 70/30 splitting.

**Figure 4:**
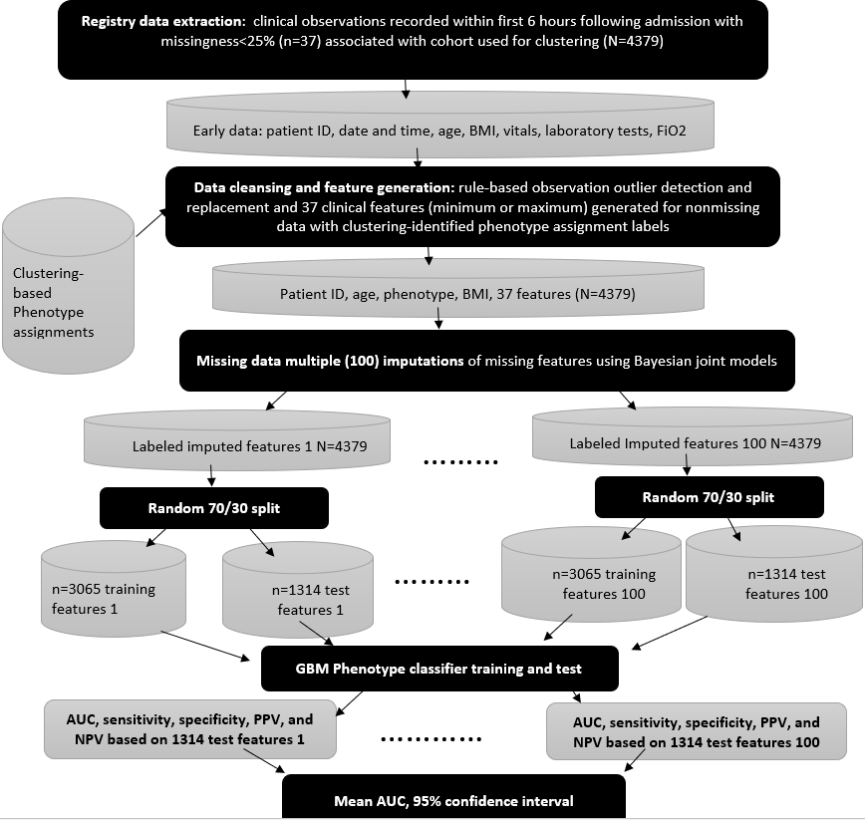
Predictive gradient-boosting machine phenotype classifier derivation process flow. AUC: area under the curve; FiO2: fraction of inspired oxygen; GBM: gradient-boosting machine; NPV: negative predictive value; PPV: positive predictive value.

The GBM was trained with 10-fold cross-validation for hyperparameter tuning using a grid search to optimize the models using the 100 training sets. Prediction performance (eg, AUC, sensitivity, and specificity) was assessed on the held-out test sets. The final performance metrics were estimated by averaging the performance estimates obtained from each imputed data set. An overview of the phenotype prediction process flow is shown in Figure 4. The classification and training R package *caret* (version 6.0-93) [61] was used for prediction phenotype classifier development.

### Ethical Considerations

This study was approved by the JH institutional review board (IRB00250903).

## Results

### Clustering and Phenotype Assignment and Associated Statistics

**Figure 5:**
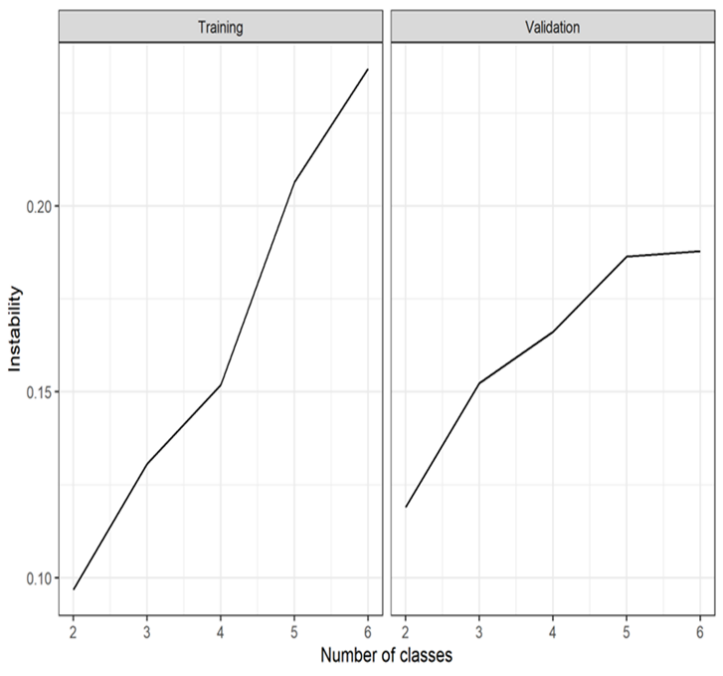
Demonstration that k=2 is the optimal number of clusters based on instability analysis for both the training and validation data sets.

By examining the total instability over different numbers of clusters [52], 2 clusters were found to be optimal in both the training and validation cohorts as, in both cases, k=2 exhibited the least instability (Figure 5). The final assignment of each patient to 1 of the 2 phenotypes in each cohort (phenotype 1: 1284/4379, 29.32% and 1258/4379, 28.73%; phenotype 2: 898/4379, 20.51% and 939/4379, 21.44% in the training and validation cohorts, respectively) was determined by NMF consensus clustering using 2 clusters.

**Figure 6:**
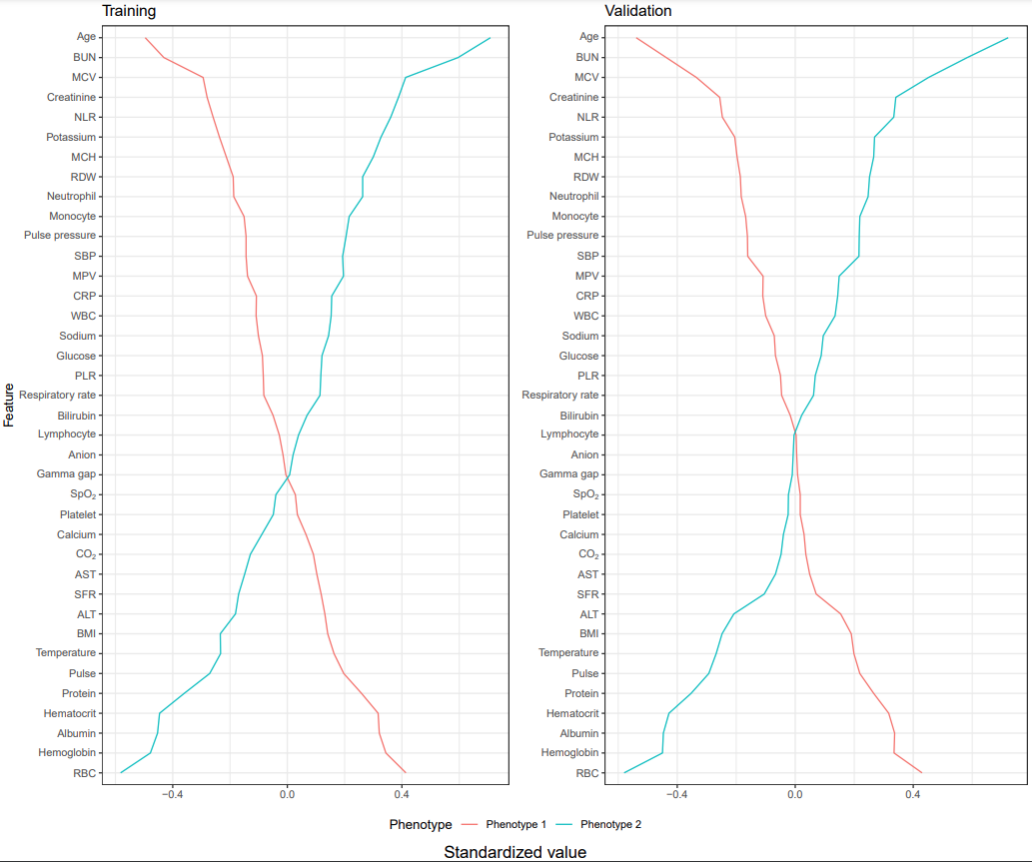
Rank plots showing agreement in the most significant phenotype-defining features (eg, age, blood urea nitrogen [BUN], mean corpuscular volume [MCV], creatinine, neutrophil-to-lymphocyte ratio [NLR], red blood cell count [RBC], hemoglobin, and hematocrit) across phenotypes in both the training and validation data sets. ALT: alanine transaminase; AST: aspartate aminotransferase; CO2: carbon dioxide; CRP: C-reactive protein; MCH: mean corpuscular hemoglobin; MPV: mean platelet volume; PLR: platelet-to-lymphocyte ratio; RDW: red cell distribution width; SBP: systolic blood pressure; SFR: oxygen saturation–to–fraction of inspired oxygen ratio; SpO2: oxygen saturation; WBC: white blood cell count.

Figure 6 depicts rank plots in which the 38 features used for training and validation cohort clustering are normalized with respect to the mean and SD of the population of the underlying paired phenotypes. Between-phenotype comparisons through nonparametric statistical methods indicate that, among the considered features in both cohorts, the most significant phenotype-defining features include age, blood urea nitrogen, creatinine, and elevated inflammatory laboratory values (neutrophils, neutrophil-to-lymphocyte ratio, red blood cell count, and albumin).

**Figure 7:**
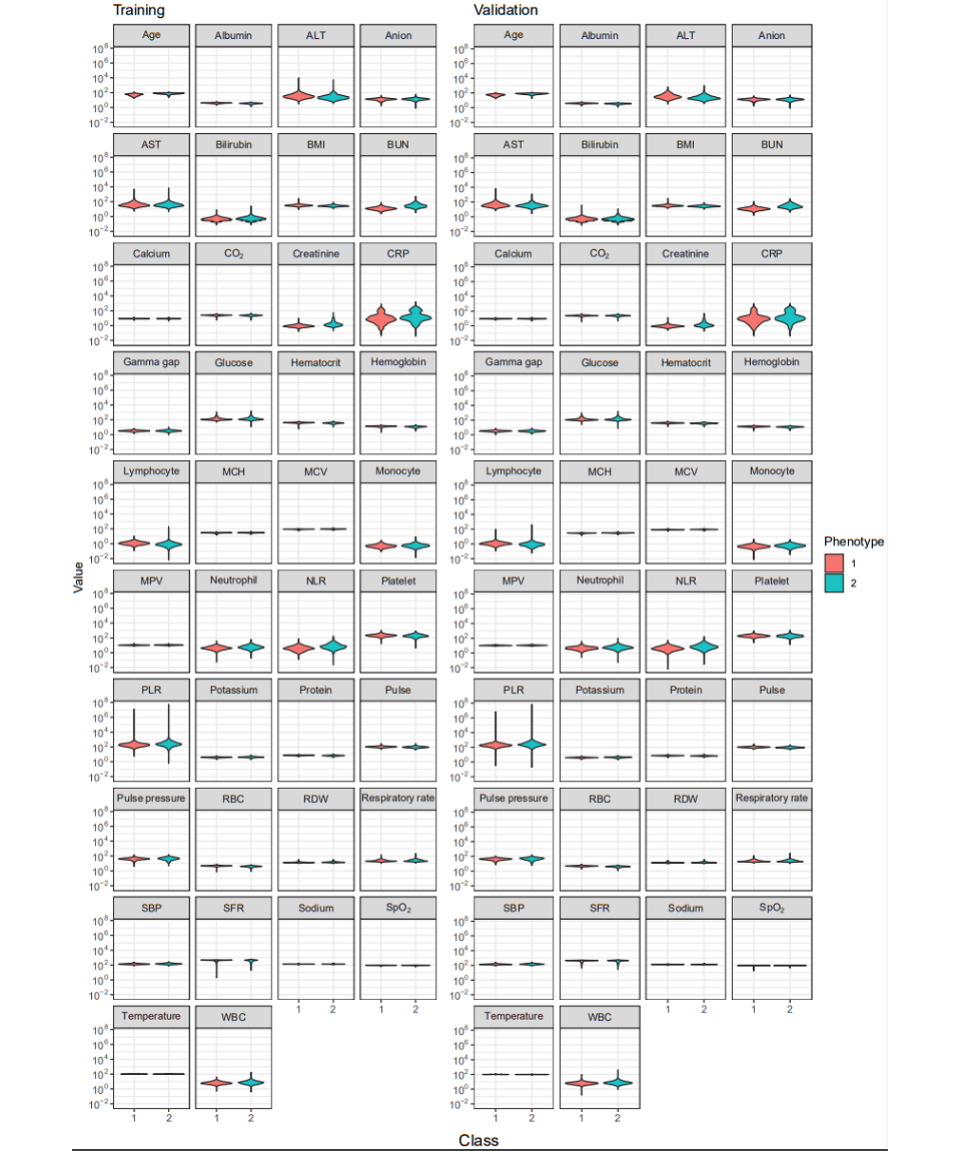
Violin plots of clustered features showing highly similar distributions and densities of features across phenotypes in both cohorts. ALT: alanine transaminase; AST: aspartate aminotransferase; BUN: blood urea nitrogen; CO2: carbon dioxide; CRP: C-reactive protein; MCH: mean corpuscular hemoglobin; MCV: mean corpuscular volume; MPV: mean platelet volume; NLR: neutrophil-to-lymphocyte ratio; PLR: platelet-to-lymphocyte ratio; RBC: red blood cell count; RDW: red cell distribution width; SBP: systolic blood pressure; SFR: oxygen saturation–to–fraction of inspired oxygen ratio; SpO2: oxygen saturation; WBC: white blood cell count.

Figure 7 depicts violin plots of the clustered training and validation data features. In this display of the summary statistics, distribution, and density of each variable, it appears that features across phenotypes share similar distributions and densities.

**Figure 8:**
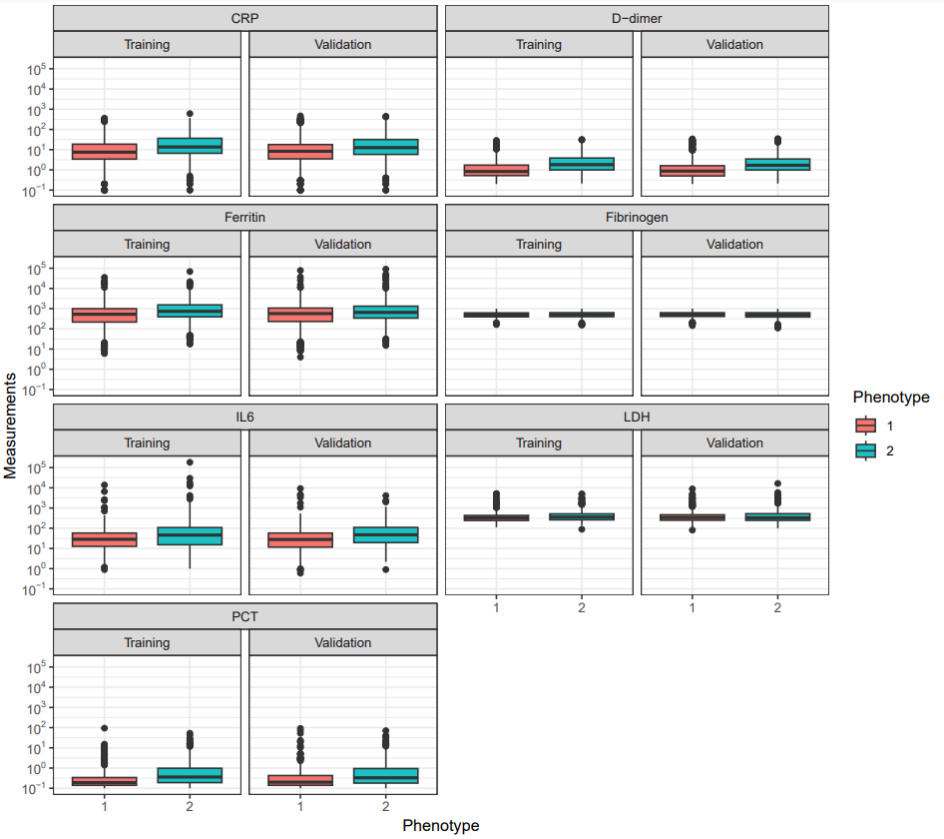
Differences in inflammatory biomarkers across phenotypes showing that phenotype 2, associated with hyperinflammatory biomarkers, was not used in clustering (D-dimer, ferritin, fibrinogen, interleukin 6 [IL6], lactate dehydrogenase [LDH], and procalcitonin [PCT]). CRP: C-reactive protein.

Figure 8 shows the differences in inflammatory biomarkers (CRP, interleukin 6, D-dimer, ferritin, lactate dehydrogenase, procalcitonin, and fibrinogen) in both cohorts associated with poor COVID-19 outcomes, as reported in previous studies [62,63]. In both the training and validation cohorts, phenotype 2 was associated with elevated inflammatory markers.

### Phenotype Association With Comorbidities and Features

**Figure 9:**
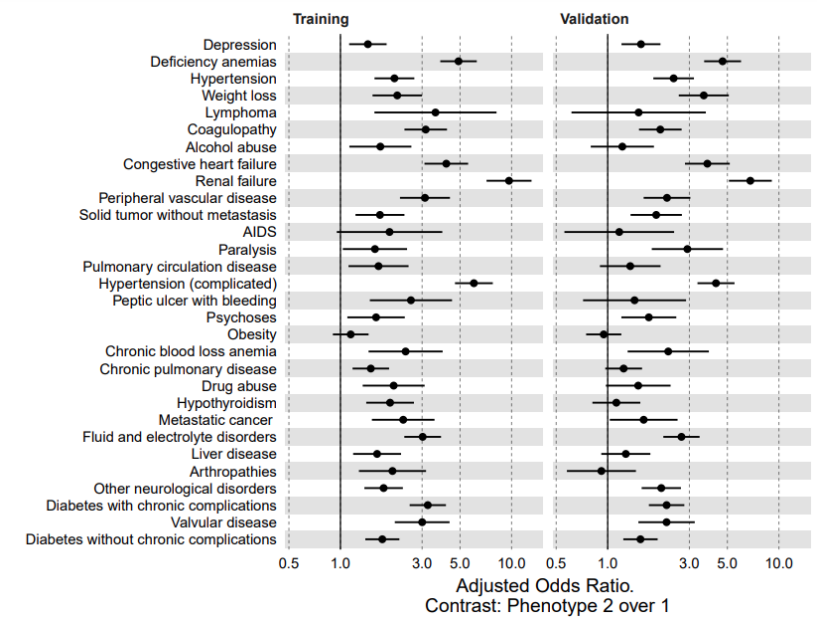
Adjusted odds ratios of comorbidities to clinical phenotypes showing similar associations between comorbidities and high severity (phenotype 2) of COVID-19 in both cohorts.

**Figure 10:**
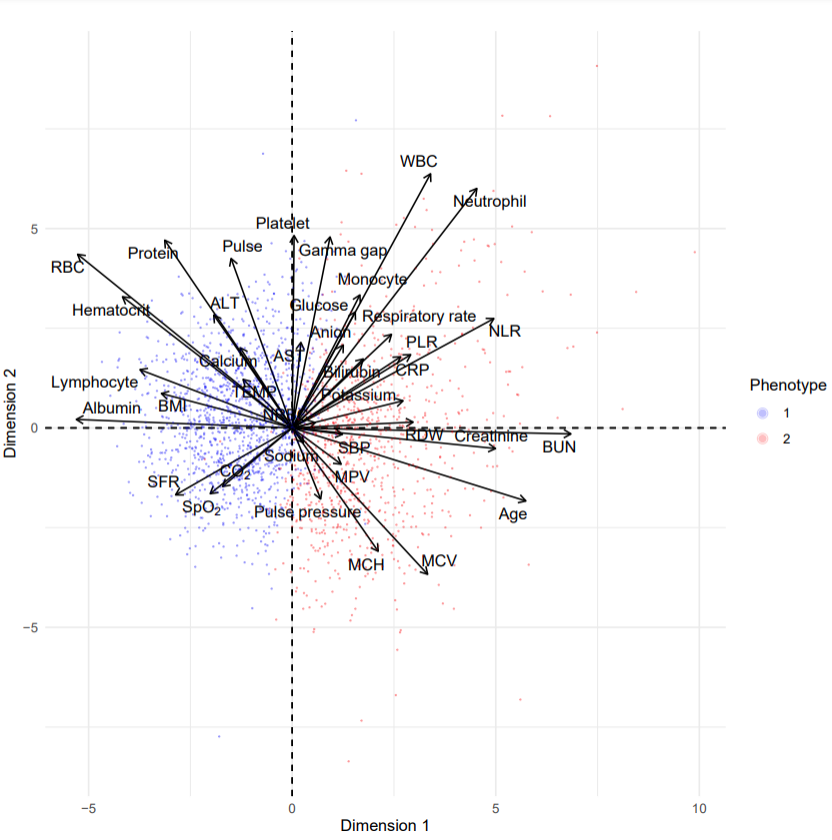
Principal-component analysis (PCA) biplot (training data) showing “good” cluster separation or spatial distribution and similar feature loading (correlations between key phenotype-defining features and principal components) with validation PCA. ALT: alanine transaminase; AST: aspartate aminotransferase; BUN: blood urea nitrogen; CO2: carbon dioxide; CRP: C-reactive protein; MCH: mean corpuscular hemoglobin; MCV: mean corpuscular volume; MPV: mean platelet volume; NLR: neutrophil-to-lymphocyte ratio; PLR: platelet-to-lymphocyte ratio; RBC: red blood cell count; RDW: red cell distribution width; Resp_rate: respiratory rate; SBP: systolic blood pressure; SFR: oxygen saturation–to–fraction of inspired oxygen ratio; SpO2: oxygen saturation; TEMP: temperature; WBC: white blood cell count.

**Figure 11:**
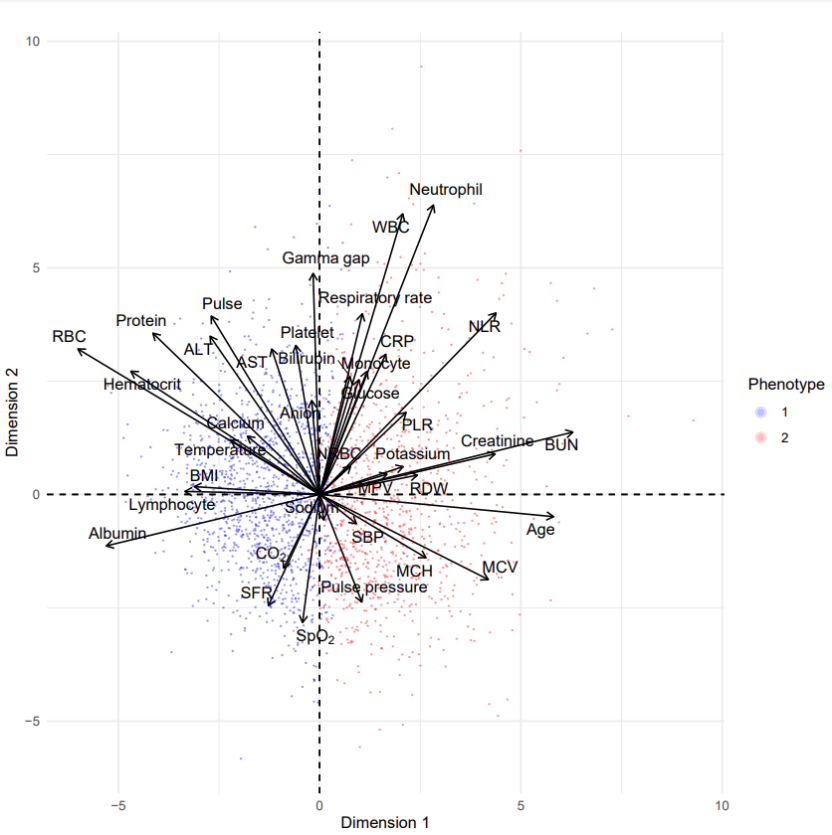
Principal-component analysis (PCA) biplot (validation data) showing “good” cluster separation or spatial distribution and similar feature loading (direction or magnitude) of correlations between key phenotype-defining features and principal components with validation PCA. ALT: alanine transaminase; AST: aspartate aminotransferase; BUN: blood urea nitrogen; CO2: carbon dioxide; CRP: C-reactive protein; MCH: mean corpuscular hemoglobin; MCV: mean corpuscular volume; MPV: mean platelet volume; NLR: neutrophil-to-lymphocyte ratio; PLR: platelet-to-lymphocyte ratio; RBC: red blood cell count; RDW: red cell distribution width; Resp_rate: respiratory rate; SBP: systolic blood pressure; SFR: oxygen saturation–to–fraction of inspired oxygen ratio; SpO2: oxygen saturation; TEMP: temperature; WBC: white blood cell count.

Table 3 and Figure 9 show the odds ratios of phenotype 2 versus phenotype 1 associated with comorbidities adjusted for age, race, gender, and ethnicity in both cohorts. These results suggest that patients in phenotype 2 have a higher likelihood of anemias, lymphoma, coagulopathy, congestive heart failure, preexisting renal failure, peripheral vascular disease, AIDS, complicated hypertension, bleeding peptic ulcers, cancer, electrolyte disorders, and diabetes with chronic complications. Figures 10 and 11 are principal-component analysis biplots including a scatterplot that shows the similarity of 2D projections of clustered observations or patients. These figures have a superimposed loading plot that shows how strongly features influence a phenotype (eg, strong associations between phenotype 1 and lymphocytes, SpO_2_/FiO_2_, and albumin and between phenotype 2 and systolic blood pressure, age, creatinine, and pulse pressure).

**Table 3:** Odds ratios (ORs) of comorbidities in phenotype 2 versus phenotype 1 adjusted for age, gender, race, and ethnicity in the training and validation cohorts^a^.

Comorbidity	Training cohort, OR (95% CI)	Validation cohort, OR (95% CI)
Depression	1.45 (1.12-1.86)	1.56 (1.20-2.03)
Deficiency anemias	4.90 (3.83-6.26)	4.69 (3.66-6.01)
Hypertension	2.07 (1.58-2.70)	2.43 (1.85-3.19)
Weight loss	2.15 (1.54-3.00)	3.64 (2.60-5.10)
Lymphoma	3.59 (1.58-8.16)	1.52 (0.61-3.74)
Coagulopathy	3.15 (2.37-4.19)	2.03 (1.52-2.70)
Alcohol abuse	1.71 (1.13-2.60)	1.22 (0.80-1.86)
Congestive heart failure	4.15 (3.10-5.57)	3.82 (2.82-5.16)
Renal failure	9.66 (7.14-13.07)	6.81 (5.10-9.09)
Peripheral vascular disease	3.12 (2.23-4.36)	2.22 (1.62-3.04)
Solid tumor without metastasis	1.70 (1.22-2.37)	1.92 (1.36-2.71)
AIDS	1.94 (0.95-3.94)	1.17 (0.56-2.44)
Paralysis	1.59 (1.03-2.45)	2.92 (1.81-4.72)
Pulmonary circulation disease	1.67 (1.11-2.50)	1.35 (0.90-2.04)
Hypertension (complicated)	6.02 (4.67-7.76)	4.29 (3.34-5.50)
Peptic ulcer with bleeding	2.58 (1.49-4.49)	1.43 (0.72-2.87)
Psychoses	1.61 (1.10-2.38)	1.74 (1.20-2.52)
Obesity	1.15 (0.90-1.46)	0.95 (0.75-1.20)
Chronic blood loss anemia	2.40 (1.46-3.95)	2.26 (1.31-3.90)
Chronic pulmonary disease	1.50 (1.18-1.92)	1.24 (0.97-1.59)
Drug abuse	2.05 (1.35-3.10)	1.51 (0.98-2.33)
Hypothyroidism	1.95 (1.42-2.69)	1.12 (0.81-1.55)
Metastatic cancer	2.33 (1.53-3.54)	1.62 (1.03-2.56)
Fluid and electrolyte disorders	3.02 (2.36-3.87)	2.70 (2.11-3.44)
Liver disease	1.64 (1.19-2.26)	1.28 (0.92-1.77)
Arthropathies	2.01 (1.28-3.16)	0.92 (0.58-1.46)
Other neurological disorders	1.79 (1.38-2.32)	2.06 (1.58-2.68)
Diabetes with chronic complications	3.24 (2.54-4.13)	2.21 (1.74-2.80)
Valvular disease	3.00 (2.08-4.34)	2.21 (1.51-3.22)
Diabetes without chronic complications	1.76 (1.40-2.21)	1.55 (1.23-1.96)

^a^Adjusted OR; contrast: phenotype 2 over phenotype 1.

### Phenotype Association With Treatments, Interventions, and Mortality

The detailed demographics, clinical characteristics, and statistical significance of the feature and outcome distribution across phenotypes in both the training and validation cohorts are shown in Tables 4 and 5. In these tables, *P* values suggest statistically significant associations between the need for ICU-level care and poor outcomes associated with phenotype 2 in both cohorts. Specifically, as shown in Table 4, phenotype-2 patients were associated with advanced age (mean 76, SD 14.2 years in phenotype 2 vs 52 years in phenotype 1) and with statistically significant (*P*<.001) increased risk of developing sepsis (34% in phenotype 2 vs 21% in phenotype 1), requiring mechanical ventilation (11% in phenotype 2 vs 4.5% in phenotype 1), using vasopressors (10.5% in phenotype 2 vs 3.5% in phenotype 1), requiring HFNC (16% in phenotype 2 vs 8% in phenotype 1), requiring continuous renal replacement therapy (CRRT; 2.4% in phenotype 2 vs 0.5% in phenotype 1), requiring dialysis (8% in phenotype 2 vs 0.6% in phenotype 1), and mortality (17% in phenotype 2 vs 2.5% in phenotype 1).

**Table 4:** Distribution of features and outcomes across phenotypes identified within the training and validation cohorts (N=4379).

Characteristic				Training cohort						Validation cohort				
				Phenotype 1 (n=1284)		Phenotype 2 (n=898)		*P* value		Phenotype 1 (n=1258)		Phenotype 2 (n=939)		*P* value
**Features, median (IQR)**														
	Age (years)		53.0 (39.3-63.5)		76.4 (66.5-85.5)		<.001		51.5 (39.4-62.1)		76.7 (65.8-85.7)		<.001	
	BMI (kg/m^2^)		30.7 (26.5-36.5)		26.6 (23.4-30.8)		<.001		31.1 (26.7-36.6)		26.9 (23.4-31.5)		<.001	
	Albumin (minimum)		4.0 (3.6-4.3)		3.5 (3.1-3.8)		<.001		3.9 (3.6-4.3)		3.5 (3.1-3.9)		<.001	
	ALT^a^ (maximum)		32.0 (21.0-52.0)		23.0 (15.0-36.0)		<.001		31.0 (21.0-52.0)		21.0 (15.0-33.0)		<.001	
	Anion (maximum)		13.0 (10.0-15.0)		13.0 (11.0-16.0)		<.001		13.0 (11.0-16.0)		13.0 (10.0-16.0)		.48	
	AST^b^ (maximum)		36.0 (26.0-55.0)		37.0 (25.0-54.5)		.36		37.0 (25.0-58.0)		32.0 (23.0-46.0)		<.001	
	Bilirubin (maximum)		0.4 (0.3-0.6)		0.5 (0.4-0.7)		<.001		0.5 (0.3-0.7)		0.5 (0.4-0.7)		.11	
	BUN^c^ (maximum)		12.0 (9.0-16.0)		27.0 (19.0-41.0)		<.001		12.0 (9.0-16.0)		26.0 (18.0-41.0)		<.001	
	Calcium (minimum)		8.8 (8.5-9.2)		8.7 (8.3-9.2)		<.001		8.8 (8.4-9.2)		8.7 (8.4-9.2)		.047	
	CO_2_^d^ (minimum)		25.0 (23.0-27.0)		23.5 (21.0-26.0)		<.001		24.0 (22.0-26.0)		24.0 (21.0-26.0)		.05	
	Creatinine (maximum)		0.9 (0.7-1.1)		1.4 (1.0-2.2)		<.001		0.9 (0.7-1.1)		1.3 (0.9-2.1)		<.001	
	CRP^e^ (maximum)		7.2 (3.2-17.9)		12.8 (6.2-32.9)		<.001		8.1 (3.3-17.4)		11.6 (5.4-30.7)		<.001	
	D-dimer (maximum)		0.6 (0.4-1.0)		1.3 (0.8-2.4)		<.001		0.7 (0.4-1.1)		1.2 (0.7-2.1)		<.001	
	Gamma gap (minimum)		3.2 (2.9-3.7)		3.3 (2.8-3.8)		.76		3.2 (2.9-3.7)		3.2 (2.8-3.7)		.36	
	Glucose (maximum)		116.0 (101.0-145.0)		125.0 (105.0-164.0)		<.001		116.0 (101.0-146.0)		123.0 (105.0-168.0)		<.001	
	Hematocrit (minimum)		41.1 (38.0-44.0)		36.5 (32.3-40.4)		.003		41.1 (38.0-44.3)		37.2 (32.9-40.7)		<.001	
	Hemoglobin (minimum)		13.4 (12.4-14.5)		11.8 (10.3-13.2)		<.001		13.5 (12.3-14.7)		12.0 (10.4-13.2)		<.001	
	Lymphocyte (minimum)		1.1 (0.8-1.6)		0.8 (0.5-1.1)		<.001		1.1 (0.8-1.5)		0.8 (0.6-1.2)		<.001	
	MCH^f^ (minimum)		28.9 (27.2-30.0)		29.7 (28.2-31.2)		<.001		28.7 (27.1-30.0)		29.7 (28.1-30.9)		<.001	
	MCV^g^ (minimum)		87.4 (83.8-90.5)		91.5 (87.5-95.8)		.06		86.8 (83.1-89.8)		91.5 (87.8-95.2)		<.001	
	Monocyte (minimum)		0.5 (0.3-0.7)		0.5 (0.4-0.8)		<.001		0.4 (0.3-0.6)		0.6 (0.4-0.8)		<.001	
	MPV^h^ (maximum)		10.3 (9.7-10.9)		10.6 (10.0-11.3)		<.001		10.3 (9.7-11.0)		10.6 (9.9-11.3)		<.001	
	Neutrophil (maximum)		4.2 (3.0-6.0)		5.3 (3.6-8.0)		<.001		4.3 (3.1-6.0)		5.2 (3.7-7.9)		<.001	
	NLR^i^ (maximum)		3.7 (2.3-5.9)		6.7 (3.9-11.4)		<.001		3.9 (2.4-6.3)		6.5 (3.8-11.1)		<.001	
	Platelet (minimum)		212.0 (167.0-266.0)		186.0 (144.0-251.0)		<.001		205.0 (160.0-262.0)		197.0 (148.2-257.0)		.01	
	PLR^j^ (maximum)		182.9 (130.2-259.2)		239.5 (153.8-372.2)		<.001		184.8 (134.2-261.0)		236.1 (162.0-376.2)		<.001	
	Potassium (maximum)		3.9 (3.6-4.2)		4.2 (3.8-4.6)		<.001		4.0 (3.7-4.3)		4.2 (3.9-4.6)		<.001	
	Protein (minimum)		7.2 (6.8-7.6)		6.7 (6.3-7.3)		<.001		7.2 (6.8-7.6)		6.8 (6.3-7.2)		<.001	
	Pulse (maximum)		102.0 (90.0-115.0)		92.0 (81.0-106.0)		<.001		102.0 (91.0-114.0)		92.0 (81.0-103.0)		<.001	
	Pulse pressure (minimum)		40.0 (33.0-49.0)		43.0 (33.0-57.0)		<.001		41.0 (33.0-49.0)		46.0 (34.0-59.0)		<.001	
	RBC^k^ (minimum)		4.7 (4.3-5.1)		4.0 (3.5-4.4)		<.001		4.8 (4.4-5.2)		4.0 (3.6-4.5)		<.001	
	RDW^l^ (maximum)		13.2 (12.5-14.1)		14.2 (13.2-15.5)		<.001		13.2 (12.5-14.3)		14.0 (13.0-15.3)		<.001	
	Respiratory rate (maximum)		20.0 (18.0-26.0)		23.0 (20.0-29.0)		<.001		20.0 (18.0-26.0)		22.0 (19.0-28.0)		.005	
	SBP^m^ (maximum)		137.0 (125.0-151.0)		145.0 (130.0-162.0)		<.001		137.0 (126.0-149.0)		146.0 (130.0-164.0)		<.001	
	SpO_2_^n^ to FiO_2_^o^ ratio (minimum)		442.9 (379.2-476.2)		428.6 (265.0-476.2)		<.001		438.1 (361.0-476.2)		433.3 (301.9-476.2)		.03	
	Sodium (minimum)		137.0 (134.0-139.0)		137.0 (134.0-140.0)		.87		137.0 (134.0-139.0)		137.0 (134.0-140.0)		.01	
	SpO_2_ (minimum)		95.0 (92.0-97.0)		94.0 (91.0-97.0)		<.001		95.0 (92.0-97.0)		95.0 (92.0-97.0)		.71	
	Temperature (maximum)		99.5 (98.6-100.9)		99.0 (98.3-100.2)		<.001		99.7 (98.7-101.3)		98.9 (98.2-100.0)		<.001	
	WBC^p^ (maximum)		6.0 (4.7-8.0)		7.0 (5.0-9.9)		<.001		6.1 (4.7-8.0)		7.0 (5.2-9.8)		<.001	
**Demographics, n (%)**														
	**Age group (years)**							<.001						<.001
		21-30	127 (9.89)		4 (0.44)				130 (10.33)		6 (0.63)			
		31-40	232 (18.06)		22 (2.44)				209 (16.61)		18 (1.91)			
		41-50	217 (16.9)		35 (3.89)				264 (20.98)		29 (3.08)			
		51-60	287 (22.35)		67 (7.46)				299 (23.76)		87 (9.26)			
		61-70	260 (20.24)		196 (21.82)				221 (17.57)		184 (19.59)			
		71-80	124 (9.65)		210 (23.38)				105 (8.34)		245 (26.09)			
		81-89	31 (2.41)		229 (25.5)				25 (1.98)		242 (25.77)			
		≥90	6 (0.46)		135 (15.03)				5 (0.39)		128 (13.63)			
	Sex (male)		566 (44.08)		481 (53.56)		<.001		630 (50.07)		464 (49.41)		.79	
	**Race**							<.001						.001
		Asian	89 (6.93)		51 (5.67)				61 (4.84)		46 (4.89)			
		Black	474 (36.91)		312 (34.74)				470 (37.36)		296 (31.52)			
		White	358 (27.88)		438 (48.77)				344 (27.34)		489 (52.08)			
		Other	358 (27.88)		88 (9.8)				375 (29.8)		105 (11.18)			
		Unknown	5 (0.38)		9 (1)				8 (0.64)		3 (0.32)			
	**Ethnicity**							<.001						<.001
		Hispanic	323 (25.16)		80 (8.91)				350 (27.82)		87 (9.27)			
		Not Hispanic	954 (74.3)		808 (89.98)				902 (71.7)		847 (90.2)			
		Patient refused	3 (0.23)		2 (0.22)				3 (0.23)		0 (0)			
		Unknown	4 (0.31)		8 (0.89)				3 (0.23)		5 (0.53)			
**Outcomes**														
	Sepsis, n (%)		257 (20.02)		306 (34.07)		<.001		273 (21.7)		315 (33.55)		<.001	
	ARDS^q^, n (%)		75 (5.84)		77 (8.57)		.02		94 (7.47)		72 (7.67)		.93	
	PEEP^r^, mean (SD)		8.5 (2.9)		7.8 (3.1)		.20		7.9 (3.1)		7.8 (2.7)		.72	
	Ventilation, n (%)		53 (4.13)		97 (10.8)		<.001		63 (5.01)		105 (11.19)		<.001	
	IV^s^ pressor, n (%)		41 (3.19)		98 (10.91)		<.001		49 (3.90)		94 (10.01)		<.001	
	ECMO^t^, n (%)		0 (0)		0 (0)		N/A^u^		1 (0.08)		0 (0)		.001	
	Death, n (%)		25 (1.95)		150 (16.70)		<.001		39 (3.10)		160 (17.04)		<.001	
	Ventilation duration (days), median (IQR)		6.6 (1.5-12.6)		8.2 (2.1-17.9)		.45		7.9 (3.3-12.5)		8.3 (2.2-14.7)		<.001	
	HFNC^v^ duration (days), median (IQR)		4.7 (2.4-8.9)		3.1 (0.9-6.5)		.03		3.4 (1.2-6.8)		3.8 (1.5-6.2)		.90	
	LOS^w^ (days), median (IQR)		3.7 (1.9-6.3)		6.0 (3.5-10.3)		<.001		3.6 (1.8-6.5)		5.8 (3.2-10.4)		<.001	
	HFNC, n (%)		99 (7.71)		155 (17.26)		<.001		124 (9.86)		139 (14.8)		<.001	
	CRRT^x^, n (%)		4 (0.31)		20 (2.23)		<.001		9 (0.72)		24 (2.56)		<.001	
	Dialysis, n (%)		3 (0.23)		84 (9.35)		<.001		12 (.95)		74 (7.88)		<.001	
	Antibiotic, n (%)		436 (33.96)		498 (55.46)		<.001		447 (35.53)		488 (51.97)		<.001	
	Anticoagulant, n (%)		31 (2.41)		21 (2.34)		<.001		24 (1.91)		23 (2.45)		<.001	
	Steroid, n (%)		74 (5.76)		75 (8.35)		.02		77 (6.12)		62 (6.60)		.71	

^a^ALT: alanine transaminase.

^b^AST: aspartate aminotransferase.

^c^BUN: blood urea nitrogen.

^d^CO_2_: carbon dioxide.

^e^CRP: C-reactive protein.

^f^MCH: mean corpuscular hemoglobin.

^g^MCV: mean corpuscular volume.

^h^MPV: mean platelet volume.

^i^NLR: neutrophil-to-lymphocyte ratio.

^j^PLR: platelet-to-lymphocyte ratio.

^k^RBC: red blood cell count.

^l^RDW: red cell distribution width.

^m^SBP: systolic blood pressure.

^n^SpO_2_: oxygen saturation.

^o^FiO_2_: fraction of inspired oxygen.

^p^WBC: white blood cell count.

^q^ARDS: acute respiratory distress syndrome.

^r^PEEP: positive end-expiratory pressure.

^s^IV: intravenous.

^t^ECMO: extracorporeal membrane oxygenation.

^u^N/A: not applicable.

^v^HFNC: high-flow nasal cannula.

^w^LOS: length of stay.

^x^CRRT: continuous renal replacement therapy.

**Table 5:** Outcomes or treatments by phenotype in the training and validation cohorts (N=4379).

Characteristic	Training cohort			Validation cohort		
	Phenotype 1 (n=1284), n (%)	Phenotype 2 (n=898), n (%)	*P* value	Phenotype 1 (n=1258), n (%)	Phenotype 2 (n=939), n (%)	*P* value
Sepsis	257 (20.02)	306 (34.08)	<.001	273 (21.7)	315 (33.55)	<.001
Ventilation	53 (4.13)	97 (10.8)	<.001	63 (5.17)	105 (11.18)	<.001
IV pressor^a^	41 (3.19)	98 (10.91)	<.001	49 (3.9)	94 (10.01)	<.001
HFNC^b^	99 (7.71)	155 (17.26)	<.001	124 (9.86)	139 (14.8)	<.001
CRRT^c^	4 (0.31)	20 (2.23)	<.001	9 (0.72)	24 (2.56)	<.001
Dialysis	3 (0.23)	84 (9.35)	<.001	12 (.95)	74 (7.88)	<.001
Death	25 (1.95)	150 (16.7)	<.001	39 (3.1)	160 (17.04)	<.001

^a^IV pressor: vasopressors administered intravenously.

^b^HFNC: high-flow nasal cannula.

^c^CRRT: continuous renal replacement therapy.

### Survival

**Figure 12:**
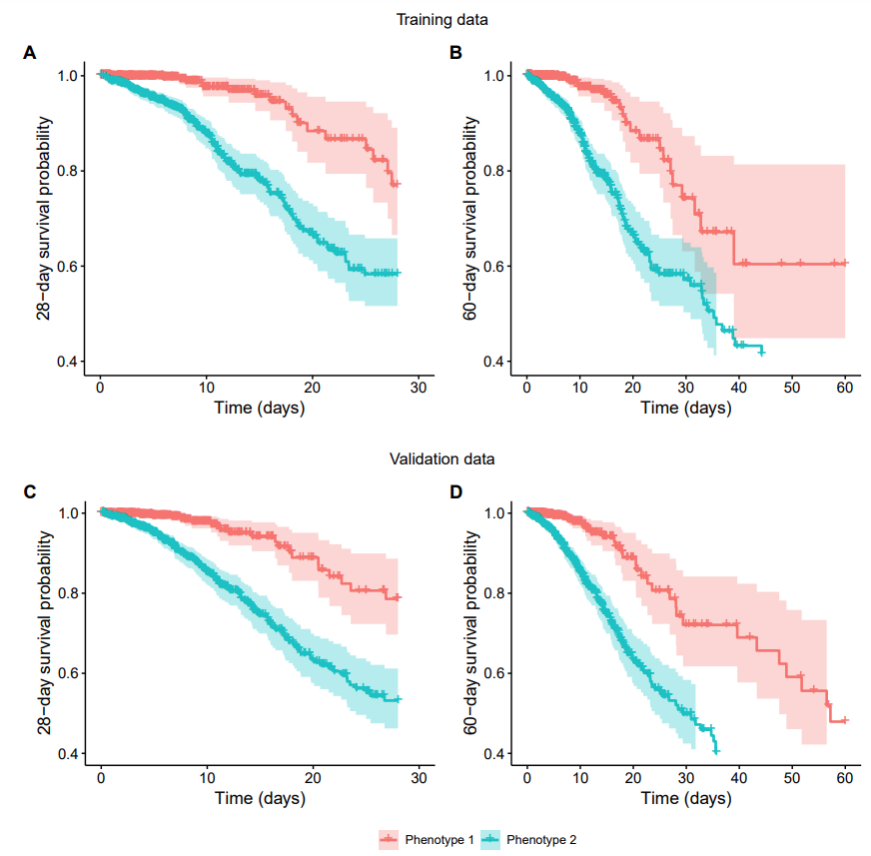
Survival curves for patients in phenotype 2 versus phenotype 1 (days) showing significantly lower survival in phenotype 2 versus phenotype 1 in both the training and validation cohorts.

Figure 12 shows survival curves. In both cohorts, survival between phenotypes diverged on day 1 from admission, and the divergence was sustained over 60 days, with significantly lower survival in the phenotype 2 group than in the phenotype 1 group.

### Prediction

The 4379 patients used for phenotype prediction analysis did not include those who initiated some form of critical care therapy (eg, HFNC or mechanical ventilation) or died within the 6 hours following admission. Clustering-identified phenotypes and clinical features (Textbox 1) based on observations recorded within the first 6 hours associated with the included patients were used for GBM predictive classifier derivation except for CRP, which was excluded because of excessive missingness (>40%). The classifier performance was based on comparing clustering-identified phenotype labels with predicted labels in the held-out test sets. Table 6 shows predictive metrics with 95% CIs of the classifier performance over the 100 imputed test sets (1314/4379, 30.01%); the mean AUC to accurately predict a test patient’s clustering-derived phenotype was at 0.89 (95% CI 0.887-0.893).

**Table 6:** Phenotype prediction model performance characteristics.

Metric	Estimate (95% CI)
Area under the curve	0.890 (0.887-0.893)
Sensitivity	0.846 (0.822-0.873)
Specificity	0.851 (0.828-0.876)
Positive predictive value	0.834 (0.907-0.865)
Negative predictive value	0.861 (0.845-0.879)

## Discussion

### Principal Findings

The key aim of our study was to develop the foundations of an EHR data-screening tool that may assist clinicians in the early identification of patients among a population of highly heterogeneous hospitalized patients with COVID-19 likely to deteriorate to hyperinflammation and require ICU-level care that may include respiratory support across a broad spectrum of modalities, such as HFNC, Nasal intermittent positive pressure ventilation, intubation or mechanical ventilation, and extracorporeal membrane oxygenation. Patients with hyperinflammation may also develop life-threatening comorbidities such as septic shock [64] and AKI [65], driving the need for specialized care, such as vasopressors [66], intermittent dialysis, and CRRT [67].

The heterogeneity of hospitalized patients with COVID-19 [68] suggests the potential benefits of clustering encounter data to identify phenotypes with distinct host response patterns to treatment that may help guide personalized therapeutics. Recent studies in Europe and the United States have identified 2 homogeneous clinical phenotypes in hospitalized patients with COVID-19 using machine learning or clustering algorithms with the potential utility to identify targeted treatment protocols [21,22].

Given that, in its most severe form, SARS-CoV-2 infections lead to life-threatening pneumonia and ARDS, clustering studies identifying phenotypes of patients with ARDS or who are mechanically ventilated and at risk of ARDS [69] are highly relevant. In total, 2 ARDS phenotypes (hyper- and hypoinflammatory) have been consistently identified in previous clustering studies [16,24,31,59,69-72] that have statistically significant similar clinical, physiological, or biomarker traits, including differential responses to treatments, interventions, and mortality rates, supporting the potential utility of machine learning–based ARDS phenotyping [27,59].

Using k-means clustering analysis of training cohort clinical data, we identified 2 distinct phenotypes that differed significantly in demographics, sepsis incidence, inflammatory biomarkers, the need for ICU-level care, and clinical outcomes including mortality. This result was reproduced in an independent clustering analysis of an internal validation cohort. These findings suggest that the early association of a new patient with a clustering-identified phenotype may provide useful prognostic information. For example, a hospitalized patient predicted to be phenotype 2 and not in current need of supplemental oxygen may be viewed as at high risk of progression to requiring ICU-level care. This patient could be flagged by hospital staff so that they might initiate close monitoring, offer empiric use of therapies such as remdesivir [73], and prepare critical care resources. Alternatively, a patient predicted to be phenotype 1 and not in current need of supplemental oxygen may be viewed as low risk, warranting supportive care only. To facilitate the early identification of patient phenotypes, this study developed a predictive GBM classifier with a mean AUC of 0.89, which would be considered an excellent statistical performance [74]. In addition, the GBM classifier used only routinely available vital signs and laboratory results observed within the first 6 hours of admission, enhancing the value of this tool as an early warning system.

To our knowledge, this is the largest clustering study identifying homogeneous phenotypes in hospitalized patients with COVID-19 using routinely available early clinical data. Independent clustering analysis of randomly selected patients in the training and validation cohorts identified a hyperinflammatory phenotype (phenotype 2) characterized by higher plasma levels of inflammatory biomarkers that were associated with a higher prevalence of HFNC, invasive mechanical ventilation, extracorporeal membrane oxygenation, CRRT, dialysis, vasopressor use, diagnosis of sepsis or ARDS, and increased mortality compared with a hypoinflammatory phenotype (phenotype 1).

Recent reports have concluded that the imbalance between hyperinflammation and immune paralysis is a hallmark of sepsis [75] and that the levels of inflammatory biomarkers such as interleukin 6 in patients with COVID-19 are associated with mortality [76]. As inherent characteristics and genetic predisposition are likely key to the heterogeneity of individual immune responses, the ability to categorize patients based on the risk of hyperinflammation allows for risk stratification and personalized treatment using targeted therapeutic regimens. The ability to identify 2 separate phenotypes based on immune condition also allows for specific treatment approaches using immunomodulators.

Our findings are in concert with those of other studies that have associated worse COVID-19 outcomes with comorbid conditions that include depression [77], anemia [78], hypertension [79], congestive heart failure [80], preexisting renal failure [81], peripheral vascular disease [82], cancer [83], paralysis or spinal cord injuries [84], chronic obstructive pulmonary disease [85], obesity [86], electrolyte imbalance [87], and diabetes [88].

Although our 2-phenotype findings are similar to those of the 2021 study of 483 patients with COVID-19 at Yale New Haven Health [21], there are several differences. In the Yale study, among the 2 identified phenotypes, the phenotype with the higher risk of mortality comprised older individuals with more comorbidities, whereas patients in the group with a lower risk of mortality comprised younger individuals who were more likely to be obese, male, and racial and ethnic minority individuals with higher levels of the CRP and ALT inflammatory markers. In contrast, our analysis identified a hyperinflammatory phenotype associated with age and comorbidities highly relevant to the development of ARDS and sepsis, both leading to increased mortality rates. However, in both the training and validation cohorts, we found that elevated ALT and BMI, male gender, and racial and ethnic minority individuals were associated with the hypoinflammatory phenotype 1 (Table 3), which is consistent with the results of the Yale study. Although the distribution of mortality between the 2 phenotypes was similar (Yale study: 25% vs 9%; this study: 23% vs 3%), the Yale study did not find statistically significant differences in the use of critical care treatments (eg, dialysis or mechanical ventilation) between the 2 phenotypes. Overall, the Yale study showed that patients who were admitted for COVID-19 were found to be classified into 2 cohorts mostly based on age-related comorbidities and specific demographics. It should be noted that both the Yale study and our study support the recent finding that, although there may be an increased incidence of severe COVID-19 among Black and Hispanic patients, this is not due to an inherent susceptibility to progression [89].

A recent systematic review of prediction models for COVID-19 [60] enumerated common weaknesses. These include a high risk of bias from inadequate sample sizes and inappropriate or incomplete evaluation of model performance with insufficient internal or external validation. In addition, calibration was often incomplete or performed using inappropriate statistics. Finally, inappropriate handling of missing data was common, including the omission of how missing data were handled. The authors summarily recommended that prediction modelers “should adhere to the TRIPOD (Transparent Reporting of a multivariate prediction model for Individual Prognosis Or Diagnosis) reporting guideline” [60]. We believe that a key strength of this study is the proactive adherence to the TRIPOD guidelines [90], thereby avoiding the weaknesses described in previous publications [91].

Our study serves as a proof of concept that combines unsupervised clustering for COVID-19 phenotype identification in historical data and supervised machine learning for phenotype prediction model derivation using routine clinical data, which is feasible as a basis for an “early warning” bedside COVID-19 screening tool [92]. If validated prospectively, such EHR data–derived and embedded models could automatically incorporate and analyze clinical data to provide real-time COVID-19 critical care decision support while minimizing disruptions to the workflow.

The prospective validation must address 2 factors. First, it is recognized that COVID-19 populations may differ significantly across time and geography with changing availability or use of vaccines, circulating SARS-CoV-2 variants, treatments, and the influence of comorbidities such as seasonal influenza and respiratory syncytial virus. Hence, the models derived in our study to identify or predict phenotypes need to be routinely “retrained” to reflect hospitalized populations with varying characteristics. With continuous access to EHR data, this issue could be addressed through machine learning models that are routinely updated with changing inpatient population characteristics.

Second, it must be prospectively demonstrated that the models can classify phenotypes robustly and consistently in real-time clinical scenarios in diverse settings. Before their clinical implementation, the models will need rigorous evaluation of their interaction with missing data frequently encountered in the real-world setting of critical care. Although we used a robust set of 38 features combined with imputation, it may be that a more effective approach would involve fewer readily available features that might predict membership to a phenotype with sufficient accuracy. The development and validation of such parsimonious models [92] require a careful analysis of the most important phenotype-defining features that would also most likely be reliably available during the early stages of an encounter. Moreover, although we used observations recorded within the first 6-hour window following admission to derive our predictive model, multisite studies have shown that the mean length of stay for patients with COVID-19 requiring ICU-level care ranges from 12 to 19 days. This suggests that predictive models trained using data over longer intervals (eg, recorded within 24 hours following admission, decreasing the prediction horizon [93]) or updating the prediction longitudinally [94] may lead to clinically useful models with improved prediction performance [95].

### Limitations

Concerning potential methodological weaknesses of this study, it should be noted that, in a head-to-head comparison of LCA versus k-means clustering in a relatively small sample of pediatric patients with sepsis (n=151), LCA was found to be somewhat more useful in identifying homogeneous phenotypes. However, both approaches identified at least one distinct high-severity phenotype [96]. Given that LCA is computationally challenging whereas k-means is better scaled to large data sets [97], most critical medicine clustering studies involving large cohorts (N>1000) in sepsis [20], ARDS [98], and COVID-19 [99,100] have effectively used k-means to identify well-separated phenotypes, leading to early detection of those who would benefit from certain treatments and close monitoring. Notably, a large cohort study by Seymour et al [20] identified and validated 4 clinical phenotypes of sepsis through k-means clustering analysis that were positively correlated with host response patterns and clinical outcomes. Most recently, Duggal et al [98] reported a k-means analysis of routine clinical data associated with a large cohort (4773 patients) that successfully identified 2 distinct ARDS phenotypes that included a phenotype with increased levels of proinflammatory markers, higher mortality, and longer duration of ventilation compared with patients in the second phenotype [99]. These studies support the validity of k-means as an effective machine learning technology for the identification of clinically useful phenotypes in “big EHR data” studies.

Another methodological weakness concerns the dependence on instability analysis (Figure 5) to identify the optimal number of phenotypes. Studies have shown that stability-based methods can be sensitive to underlying data distributions and may not always provide a valid and meaningful choice of the optimal number of k-means–derived clusters [101]. Although instability-based methods compare favorably with commonly used distance-based methods to identify the optimal k (eg, elbow and silhouette), alternatives such as the Calinski-Harabasz [102] evaluation metric that measures the compactness and separation of clusters, thereby providing a measure of the quality of the clustering results, would be a useful addition to the analysis. As this metric can be sensitive to the density and shape of clusters, in future studies, it may be beneficial to consider both stability and evaluation metrics when selecting an optimal k [103]. However, the validity of our finding that k=2 identifies the true number of COVID-19 phenotypes is bolstered by our use of a recently improved instability metric that corrects for the distribution of cluster sizes [52] and the fact that independent studies in other related populations (ARDS and other populations with COVID-19) have also identified 2 phenotypes using totally different clustering algorithms (eg, LCA).

### Conclusions

In summary, k-means clustering was effective in identifying phenotypes with distinct treatments or intervention responses and outcomes in a large cohort of hospitalized patients with COVID-19. In addition, a GBM machine learning classifier model using readily available early encounter data accurately assigned patients to phenotypes, suggesting that the application of these models in a clinical setting may provide valuable prognostic information that could inform personalized COVID-19 management. Although future studies and trials are needed to validate the clinical utility of phenotype assignment, it would seem reasonable to implement successfully validated machine learning algorithms in extant EHR systems as a tool to support those trials.

## Abbreviations

AKI: acute kidney injury
ALT: alanine aminotransferase
ARDS: acute respiratory distress syndrome
AUC: area under the curve
CRP: C-reactive protein
CRRT: continuous renal replacement therapy
EHR: electronic health record
FiO_2_: fraction of inspired oxygen
FMI: fraction of missing information
GBM: gradient-boosting machine
HFNC: high-flow nasal cannula
ICU: intensive care unit
JH: Johns Hopkins
LCA: latent class analysis
MI: multiple imputation
NMF: nonnegative matrix factorization
SpO_2_: oxygen saturation
TRIPOD: Transparent Reporting of a Multivariable Prediction Model for Individual Prognosis or Diagnosis

## Data Availability

The data sets generated and analyzed during this study are not publicly available as access to the JH-CROWN Registry requires permission from the Johns Hopkins University institutional review board, Biostatistics, Epidemiology and Data Management Core, and Institute for Clinical and Translational Research but are available from the corresponding author upon reasonable request.

## References

[1] Wang D, Hu B, Hu C, Zhu F, Liu X, Zhang J, Wang B, Xiang H, Cheng Z, Xiong Y, Zhao Y, Li Y, Wang X, Peng Z (2020). Clinical characteristics of 138 hospitalized patients with 2019 novel coronavirus-infected pneumonia in Wuhan, China. JAMA.

[2] Yang X, Yu Y, Xu J, Shu H, Xia J, Liu H, Wu Y, Zhang L, Yu Z, Fang M, Yu T, Wang Y, Pan S, Zou X, Yuan S, Shang Y (2020). Clinical course and outcomes of critically ill patients with SARS-CoV-2 pneumonia in Wuhan, China: a single-centered, retrospective, observational study. Lancet Respir Med.

[3] Cidade JP, Coelho L, Costa V, Morais R, Moniz P, Morais L, Fidalgo P, Tralhão A, Paulino C, Nora D, Valério B, Mendes V, Tapadinhas C, Povoa P (2022). Septic shock 3.0 criteria application in severe COVID-19 patients: an unattended sepsis population with high mortality risk. World J Crit Care Med.

[4] González J, Benítez ID, de Gonzalo-Calvo D, Torres G, de Batlle J, Gómez S, Moncusí-Moix A, Carmona P, Santisteve S, Monge A, Gort-Paniello C, Zuil M, Cabo-Gambín R, Manzano Senra C, Vengoechea Aragoncillo JJ, Vaca R, Minguez O, Aguilar M, Ferrer R, Ceccato A, Fernández L, Motos A, Riera J, Menéndez R, Garcia-Gasulla D, Peñuelas O, Labarca G, Caballero J, Barberà C, Torres A, Barbé F, CIBERESUCICOVID Project (COV20/00110‚ ISCIII) (2022). Impact of time to intubation on mortality and pulmonary sequelae in critically ill patients with COVID-19: a prospective cohort study. Crit Care.

[5] Colon Hidalgo D, Patel J, Masic D, Park D, Rech MA (2020). Delayed vasopressor initiation is associated with increased mortality in patients with septic shock. J Crit Care.

[6] Kolhe NV, Fluck RJ, Selby NM, Taal MW (2020). Acute kidney injury associated with COVID-19: a retrospective cohort study. PLoS Med.

[7] Chan L, Chaudhary K, Saha A, Chauhan K, Vaid A, Zhao S, Paranjpe I, Somani S, Richter F, Miotto R, Lala A, Kia A, Timsina P, Li L, Freeman R, Chen R, Narula J, Just AC, Horowitz C, Fayad Z, Cordon-Cardo C, Schadt E, Levin MA, Reich DL, Fuster V, Murphy B, He JC, Charney AW, Böttinger EP, Glicksberg BS, Coca SG, Nadkarni GN, on behalf of the Mount Sinai COVID Informatics Center (MSCIC) (2021). AKI in hospitalized patients with COVID-19. J Am Soc Nephrol.

[8] Kiekkas P, Tzenalis A, Gklava V, Stefanopoulos N, Voyagis G, Aretha D (2022). Delayed admission to the intensive care unit and mortality of critically ill adults: systematic review and meta-analysis. Biomed Res Int.

[9] Gentleman R, Carey VJ (2008). Unsupervised machine learning. Bioconductor Case Studies. Use R!.

[10] Loftus TJ, Shickel B, Balch JA, Tighe PJ, Abbott KL, Fazzone B, Anderson EM, Rozowsky J, Ozrazgat-Baslanti T, Ren Y, Berceli SA, Hogan WR, Efron PA, Moorman JR, Rashidi P, Upchurch GR, Bihorac A (2022). Phenotype clustering in health care: a narrative review for clinicians. Front Artif Intell.

[11] Castela Forte J, Perner A, van der Horst IC (2019). The use of clustering algorithms in critical care research to unravel patient heterogeneity. Intensive Care Med.

[12] Lanza ST, Rhoades BL (2013). Latent class analysis: an alternative perspective on subgroup analysis in prevention and treatment. Prev Sci.

[13] Sinha P, Calfee CS, Delucchi KL (2021). Practitioner's guide to latent class analysis: methodological considerations and common pitfalls. Crit Care Med.

[14] Yan S, Kwan YH, Tan CS, Thumboo J, Low LL (2018). A systematic review of the clinical application of data-driven population segmentation analysis. BMC Med Res Methodol.

[15] Grant RW, McCloskey J, Hatfield M, Uratsu C, Ralston JD, Bayliss E, Kennedy CJ (2020). Use of latent class analysis and k-Means clustering to identify complex patient profiles. JAMA Netw Open.

[16] Sinha P, Delucchi KL, Thompson BT, McAuley DF, Matthay MA, Calfee CS, NHLBI ARDS Network (2018). Latent class analysis of ARDS subphenotypes: a secondary analysis of the statins for acutely injured lungs from sepsis (SAILS) study. Intensive Care Med.

[17] Wilson JG, Calfee CS (2020). ARDS subphenotypes: understanding a heterogeneous syndrome. Crit Care.

[18] Famous KR, Delucchi K, Ware LB, Kangelaris KN, Liu KD, Thompson BT, Calfee CS (2017). Acute respiratory distress syndrome subphenotypes respond differently to randomized fluid management strategy. Am J Respir Crit Care Med.

[19] Gårdlund B, Dmitrieva NO, Pieper CF, Finfer S, Marshall JC, Taylor Thompson B (2018). Six subphenotypes in septic shock: latent class analysis of the PROWESS Shock study. J Crit Care.

[20] Seymour CW, Kennedy JN, Wang S, Chang C-C, Elliott CF, Xu Z, Berry S, Clermont G, Cooper G, Gomez H, Huang DT, Kellum JA, Mi Q, Opal SM, Talisa V, van der Poll T, Visweswaran S, Vodovotz Y, Weiss JC, Yealy DM, Yende S, Angus DC (2019). Derivation, validation, and potential treatment implications of novel clinical phenotypes for sepsis. JAMA.

[21] Teng C, Thampy U, Bae JY, Cai P, Dixon RA, Liu Q, Li P (2021). Identification of phenotypes among COVID-19 patients in the United States using latent class analysis. Infect Drug Resist.

[22] Gutiérrez-Gutiérrez B, Del Toro MD, Borobia AM, Carcas A, Jarrín I, Yllescas M, Ryan P, Pachón J, Carratalà J, Berenguer J, Arribas JR, Rodríguez-Baño J, REIPI-SEIMC COVID-19 groupCOVID@HULP groups (2021). Identification and validation of clinical phenotypes with prognostic implications in patients admitted to hospital with COVID-19: a multicentre cohort study. Lancet Infect Dis.

[23] Natekin A, Knoll A (2013). Gradient boosting machines, a tutorial. Front Neurorobot.

[24] Sinha P, Churpek MM, Calfee CS (2020). Machine learning classifier models can identify acute respiratory distress syndrome phenotypes using readily available clinical data. Am J Respir Crit Care Med.

[25] Churpek MM, Yuen TC, Winslow C, Meltzer DO, Kattan MW, Edelson DP (2016). Multicenter comparison of machine learning methods and conventional regression for predicting clinical deterioration on the wards. Crit Care Med.

[26] Ayaru L, Ypsilantis P-P, Nanapragasam A, Choi RC, Thillanathan A, Min-Ho L, Montana G (2015). Prediction of outcome in acute lower gastrointestinal bleeding using gradient boosting. PLoS One.

[27] Maddali MV, Churpek M, Pham T, Rezoagli E, Zhuo H, Zhao W, He J, Delucchi KL, Wang C, Wickersham N, McNeil JB, Jauregui A, Ke S, Vessel K, Gomez A, Hendrickson CM, Kangelaris KN, Sarma A, Leligdowicz A, Liu KD, Matthay MA, Ware LB, Laffey JG, Bellani G, Calfee CS, Sinha P, LUNG SAFE Investigatorsthe ESICM Trials Group (2022). Validation and utility of ARDS subphenotypes identified by machine-learning models using clinical data: an observational, multicohort, retrospective analysis. Lancet Respir Med.

[28] Bos LD, Sjoding M, Sinha P, Bhavani SV, Lyons PG, Bewley AF, Botta M, Tsonas AM, Serpa Neto A, Schultz MJ, Dickson RP, Paulus F, PRoVENT-COVID collaborative group (2021). Longitudinal respiratory subphenotypes in patients with COVID-19-related acute respiratory distress syndrome: results from three observational cohorts. Lancet Respir Med.

[29] Meyer NJ, Gattinoni L, Calfee CS (2021). Acute respiratory distress syndrome. Lancet.

[30] Shankar-Hari M, McAuley DF (2017). Acute respiratory distress syndrome phenotypes and identifying treatable traits. The dawn of personalized medicine for ARDS. Am J Respir Crit Care Med.

[31] Calfee CS, Delucchi K, Parsons PE, Thompson BT, Ware LB, Matthay MA, NHLBI ARDS Network (2014). Subphenotypes in acute respiratory distress syndrome: latent class analysis of data from two randomised controlled trials. Lancet Respir Med.

[32] COVID-19 precision medicine analytics platform registry (JH-Crown). Johns Hopkins Institute for Clinical & Translational Research.

[33] Pandya D, Nagrajappa AK, Ravi KS (2016). Assessment and correlation of urea and creatinine levels in saliva and serum of patients with chronic kidney disease, diabetes and hypertension– a research study. J Clin Diagnos Res.

[34] Doig K, Zhang B (2017). A methodical approach to interpreting the red blood cell parameters of the complete blood count. Clin Lab Sci.

[35] Ullmann T, Hennig C, Boulesteix A-L (2021). Validation of cluster analysis results on validation data: a systematic framework. WIREs Data Mining Knowl.

[36] Paxton C, Niculescu-Mizil A, Saria S (2013). Developing predictive models using electronic medical records: challenges and pitfalls. AMIA Annu Symp Proc.

[37] Lanzani C, Simonini M, Arcidiacono T, Messaggio E, Bucci R, Betti P, Avino M, Magni G, Maggioni C, Conte C, Querini PR, Ciceri F, Castagna A, Vezzoli G, Manunta P, Bio Angels for COVID-BioB Study Group (2021). Role of blood pressure dysregulation on kidney and mortality outcomes in COVID-19. Kidney, blood pressure and mortality in SARS-CoV-2 infection. J Nephrol.

[38] Nasal cannula FiO₂ estimation. Calculate by QxMD.

[39] Nowak-Brzezińska A, Gaibei I (2022). How the outliers influence the quality of clustering?. Entropy (Basel).

[40] Genes N, Chandra D, Ellis S, Baumlin K (2013). Validating emergency department vital signs using a data quality engine for data warehouse. Open Med Inform J.

[41] Outliers. University of Florida Health.

[42] Madley-Dowd P, Hughes R, Tilling K, Heron J (2019). The proportion of missing data should not be used to guide decisions on multiple imputation. J Clin Epidemiol.

[43] Audigier V, Niang N, Resche-Rigon M (2023). Clustering with missing data: which imputation model for which cluster analysis method?. arXiv. Preprint posted online June 8, 2021.

[44] Murray JS, Reiter JP (2023). Multiple imputation of missing categorical and continuous values via bayesian mixture models with local dependence. arXiv. Preprint posted online October 2, 2014.

[45] Kim HJ, Reiter JP, Wang Q, Cox LH, Karr AF (2014). Multiple imputation of missing or faulty values under linear constraints. J Bus Econ Stat.

[46] Rubin DB (1987). Multiple Imputation for Nonresponse in Surveys.

[47] von Hippel PT (2018). How many imputations do you need? A two-stage calculation using a quadratic rule. Sociol Methods Res.

[48] Von Hippel P (2019). How many imputations do you need?. Statistical Horizons.

[49] Sidky H, Young JC, Girvin AT, Lee E, Shao YR, Hotaling N, Michael S, Wilkins KJ, Setoguchi S, Funk MJ, N3C Consortium (2023). Data quality considerations for evaluating COVID-19 treatments using real world data: learnings from the National COVID Cohort Collaborative (N3C). BMC Med Res Methodol.

[50] Mourer A, Forest F, Lebbah M, Azzag H, Lacaille J (2023). Selecting the number of clusters K with a stability trade-off: an internal validation criterion. arXiv. Preprint posted online June 15, 2020.

[51] Yu H, Chapman B, Di Florio A, Eischen E, Gotz D, Jacob M, Blair RH (2018). Bootstrapping estimates of stability for clusters, observations and model selection. Comput Stat.

[52] Haslbeck JM, Wulff DU (2020). Estimating the number of clusters via a corrected clustering instability. Comput Stat.

[53] Fang Y, Wang J (2012). Selection of the number of clusters via the bootstrap method. Comput Stat Data Ana.

[54] Li T, Ding C, Jordan MI (2007). Solving consensus and semi-supervised clustering problems using nonnegative matrix factorization. Proceedings of the Seventh IEEE International Conference on Data Mining (ICDM 2007).

[55] Khan I, Luo Z (2018). Nonnegative matrix factorization based consensus for clusterings with a variable number of clusters. IEEE Access.

[56] Tibshirani R (2018). Regression shrinkage and selection via the lasso. J R Stat Soc Series B Stat Methodol.

[57] Audigier V, Niang N (2023). Clustering with missing data: which equivalent for Rubin's rules?. arXiv. Preprint posted online November 27, 2020.

[58] Kruskal-Wallis Test. Statistics Solutions.

[59] Matthay MA, Arabi YM, Siegel ER, Ware LB, Bos LD, Sinha P, Beitler JR, Wick KD, Curley MA, Constantin J, Levitt JE, Calfee CS (2020). Phenotypes and personalized medicine in the acute respiratory distress syndrome. Intensive Care Med.

[60] Wynants L, van Calster B, Collins GS, Riley RD, Heinze G, Schuit E, Bonten MM, Dahly DL, Damen JA, Debray TP, de Jong VM, De Vos M, Dhiman P, Haller MC, Harhay MO, Henckaerts L, Heus P, Kammer M, Kreuzberger N, Lohmann A, Luijken K, Ma J, Martin GP, McLernon DJ, Andaur Navarro CL, Reitsma JB, Sergeant JC, Shi C, Skoetz N, Smits LJ, Snell KI, Sperrin M, Spijker R, Steyerberg EW, Takada T, Tzoulaki I, van Kuijk SM, van Bussel B, van der Horst IC, van Royen FS, Verbakel JY, Wallisch C, Wilkinson J, Wolff R, Hooft L, Moons KG, van Smeden M (2020). Prediction models for diagnosis and prognosis of covid-19: systematic review and critical appraisal. BMJ.

[61] Kuhn M (2008). Building predictive models in R using the caret package. J Stat Softw.

[62] Suri A, Singh NK, Perumal V (2022). Association of inflammatory biomarker abnormalities with mortality in COVID-19: a meta-analysis. Bull Natl Res Cent.

[63] Stoeckle K, Witting B, Kapadia S, An A, Marks K (2022). Elevated inflammatory markers are associated with poor outcomes in COVID-19 patients treated with remdesivir. J Med Virol.

[64] Abumayyaleh M, Nuñez-Gil IJ, El-Battrawy I, Estrada V, Becerra-Muñoz VM, Uribarri A, Fernández-Rozas I, Feltes G, Arroyo-Espliguero R, Trabattoni D, López Pais J, Pepe M, Romero R, Ortega-Armas ME, Bianco M, Astrua TC, D'Ascenzo F, Fabregat-Andres O, Ballester A, Marín F, Buonsenso D, Sanchez-Gimenez R, Weiß C, Fernandez Perez C, Fernández-Ortiz A, Macaya C, Akin I (2021). Sepsis of patients infected by SARS-CoV-2: real-world experience from the international HOPE-COVID-19-registry and validation of HOPE sepsis score. Front Med (Lausanne).

[65] Marques F, Gameiro J, Oliveira J, Fonseca JA, Duarte I, Bernardo J, Branco C, Costa C, Carreiro C, Braz S, Lopes JA (2021). Acute kidney disease and mortality in acute kidney injury patients with COVID-19. J Clin Med.

[66] Russell JA (2006). Management of sepsis. N Engl J Med.

[67] Paramitha MP, Suyanto JC, Puspitasari S (2021). The role of continuous renal replacement therapy (Crrt) in Coronavirus disease 2019 (Covid-19) patients. Trends Anaesthesia Crit Care.

[68] Potere N, Valeriani E, Candeloro M, Tana M, Porreca E, Abbate A, Spoto S, Rutjes AW, Di Nisio M (2020). Acute complications and mortality in hospitalized patients with coronavirus disease 2019: a systematic review and meta-analysis. Crit Care.

[69] Kitsios GD, Yang L, Manatakis DV, Nouraie M, Evankovich J, Bain W, Dunlap DG, Shah F, Barbash IJ, Rapport SF, Zhang Y, DeSensi RS, Weathington NM, Chen BB, Ray P, Mallampalli RK, Benos PV, Lee JS, Morris A, McVerry BJ (2019). Host-response subphenotypes offer prognostic enrichment in patients with or at risk for acute respiratory distress syndrome. Crit Care Med.

[70] Calfee CS, Delucchi KL, Sinha P, Matthay MA, Hackett J, Shankar-Hari M, McDowell C, Laffey JG, O'Kane CM, McAuley DF, Irish Critical Care Trials Group (2018). Acute respiratory distress syndrome subphenotypes and differential response to simvastatin: secondary analysis of a randomised controlled trial. Lancet Respir Med.

[71] Bos LD, Schouten LR, van Vught LA, Wiewel MA, Ong DS, Cremer O, Artigas A, Martin-Loeches I, Hoogendijk AJ, van der Poll T, Horn J, Juffermans N, Calfee CS, Schultz MJ, MARS consortium (2017). Identification and validation of distinct biological phenotypes in patients with acute respiratory distress syndrome by cluster analysis. Thorax.

[72] Sinha P, Delucchi KL, McAuley DF, O'Kane CM, Matthay MA, Calfee CS (2020). Development and validation of parsimonious algorithms to classify acute respiratory distress syndrome phenotypes: a secondary analysis of randomised controlled trials. Lancet Respiratory Med.

[73] Beigel JH, Tomashek KM, Dodd LE, Mehta AK, Zingman BS, Kalil AC, Hohmann E, Chu HY, Luetkemeyer A, Kline S, Lopez de Castilla D, Finberg RW, Dierberg K, Tapson V, Hsieh L, Patterson TF, Paredes R, Sweeney DA, Short WR, Touloumi G, Lye DC, Ohmagari N, Oh M, Ruiz-Palacios GM, Benfield T, Fätkenheuer G, Kortepeter MG, Atmar RL, Creech CB, Lundgren J, Babiker AG, Pett S, Neaton JD, Burgess TH, Bonnett T, Green M, Makowski M, Osinusi A, Nayak S, Lane HC, ACTT-1 Study Group Members (2020). Remdesivir for the Treatment of Covid-19 - Final Report. N Engl J Med.

[74] Mandrekar JN (2010). Receiver operating characteristic curve in diagnostic test assessment. J Thorac Oncol.

[75] van der Poll T, Shankar-Hari M, Wiersinga WJ (2021). The immunology of sepsis. Immunity.

[76] Santa Cruz A, Mendes-Frias A, Oliveira AI, Dias L, Matos AR, Carvalho A, Capela C, Pedrosa J, Castro AG, Silvestre R (2021). Interleukin-6 is a biomarker for the development of fatal severe acute respiratory syndrome coronavirus 2 pneumonia. Front Immunol.

[77] Wang Y, Yang Y, Ren L, Shao Y, Tao W, Dai X (2021). Preexisting mental disorders increase the risk of COVID-19 infection and associated mortality. Front Public Health.

[78] Oh SM, Skendelas JP, Macdonald E, Bergamini M, Goel S, Choi J, Segal KR, Vivek K, Nair S, Leff J (2021). On-admission anemia predicts mortality in COVID-19 patients: a single center, retrospective cohort study. Am J Emerg Med.

[79] (2022). Hypertension elevates risk for more severe COVID-19 illness. Cedars Sinai.

[80] Shaw ML (2020). Prognosis poor for patients with heart failure, COVID-19. AJMC.

[81] National Kidney Foundation.

[82] Smolderen KG, Lee M, Arora T, Simonov M, Mena-Hurtado C (2022). Peripheral artery disease and COVID-19 outcomes: insights from the Yale DOM-CovX registry. Curr Probl Cardiol.

[83] Howell MD, Donnino M, Clardy P, Talmor D, Shapiro NI (2007). Occult hypoperfusion and mortality in patients with suspected infection. Intensive Care Med.

[84] Powell J (2021). People with spinal cord injuries are at risk for more challenges with COVID-19, data shows. KXAN.

[85] Gerayeli FV, Milne S, Cheung C, Li X, Yang CW, Tam A, Choi LH, Bae A, Sin DD (2021). COPD and the risk of poor outcomes in COVID-19: a systematic review and meta-analysis. EClinicalMedicine.

[86] Kompaniyets L, Goodman AB, Belay B, Freedman DS, Sucosky MS, Lange SJ, Gundlapalli AV, Boehmer TK, Blanck HM (2021). Body mass index and risk for COVID-19-related hospitalization, intensive care unit admission, invasive mechanical ventilation, and death - United States, March-December 2020. MMWR Morb Mortal Wkly Rep.

[87] De Carvalho H, Richard MC, Chouihed T, Goffinet N, Le Bastard Q, Freund Y, Kratz A, Dubroux M, Masson D, Figueres L, Montassier E (2021). Electrolyte imbalance in COVID-19 patients admitted to the Emergency Department: a case-control study. Intern Emerg Med.

[88] Izzi-Engbeaya C, Distaso W, Amin A, Yang W, Idowu O, Kenkre JS, Shah RJ, Woin E, Shi C, Alavi N, Bedri H, Brady N, Blackburn S, Leczycka M, Patel S, Sokol E, Toke-Bjolgerud E, Qayum A, Abdel-Malek M, Hope DC, Oliver NS, Bravis V, Misra S, Tan TM, Hill NE, Salem V (2021). Adverse outcomes in COVID-19 and diabetes: a retrospective cohort study from three London teaching hospitals. BMJ Open Diabetes Res Care.

[89] Shortreed SM, Gray R, Akosile MA, Walker RL, Fuller S, Temposky L, Fortmann SP, Albertson-Junkans L, Floyd JS, Bayliss EA, Harrington LB, Lee MH, Dublin S (2023). Increased COVID-19 infection risk drives racial and ethnic disparities in severe COVID-19 outcomes. J Racial Ethn Health Disparities.

[90] Collins GS, Reitsma JB, Altman DG, Moons KG (2015). Transparent reporting of a multivariable prediction model for individual prognosis or diagnosis (TRIPOD): the TRIPOD Statement. BMC Med.

[91] Su Y, Ju M, Xie R, Yu S, Zheng J, Ma G, Liu K, Ma J, Yu K, Tu G, Luo Z (2020). Prognostic accuracy of early warning scores for clinical deterioration in patients with COVID-19. Front Med (Lausanne).

[92] Murri R, Lenkowicz J, Masciocchi C, Iacomini C, Fantoni M, Damiani A, Marchetti A, Sergi PD, Arcuri G, Cesario A, Patarnello S, Antonelli M, Bellantone R, Bernabei R, Boccia S, Calabresi P, Cambieri A, Cauda R, Colosimo C, Crea F, De Maria R, De Stefano V, Franceschi F, Gasbarrini A, Parolini O, Richeldi L, Sanguinetti M, Urbani A, Zega M, Scambia G, Valentini V, Gemelli against Covid Group (2021). A machine-learning parsimonious multivariable predictive model of mortality risk in patients with Covid-19. Sci Rep.

[93] Zargoush M, Sameh A, Javadi M, Shabani S, Ghazalbash S, Perri D (2021). The impact of recency and adequacy of historical information on sepsis predictions using machine learning. Sci Rep.

[94] Wongvibulsin S, Garibaldi BT, Antar AA, Wen J, Wang M, Gupta A, Bollinger R, Xu Y, Wang K, Betz JF, Muschelli J, Bandeen-Roche K, Zeger SL, Robinson ML (2021). Development of severe COVID-19 adaptive risk predictor (SCARP), a calculator to predict severe disease or death in hospitalized patients with COVID-19. Ann Intern Med.

[95] Galanter W, Rodríguez-Fernández JM, Chow K, Harford S, Kochendorfer KM, Pishgar M, Theis J, Zulueta J, Darabi H (2021). Predicting clinical outcomes among hospitalized COVID-19 patients using both local and published models. BMC Med Inform Decis Mak.

[96] Koutroulis I, Velez T, Wang T, Yohannes S, Galarraga JE, Morales JA, Freishtat RJ, Chamberlain JM (2022). Pediatric sepsis phenotypes for enhanced therapeutics: an application of clustering to electronic health records. J Am Coll Emerg Physicians Open.

[97] (2020). Unsupervised learning with k-means clustering with large datasets. ODSC - Open Data Science.

[98] Duggal A, Kast R, van Ark E, Bulgarelli L, Siuba MT, Osborn J, Rey DA, Zampieri FG, Cavalcanti AB, Maia I, Paisani DM, Laranjeira LN, Serpa Neto A, Deliberato RO (2022). Identification of acute respiratory distress syndrome subphenotypes de novo using routine clinical data: a retrospective analysis of ARDS clinical trials. BMJ Open.

[99] Abdullah D, Susilo S, Ahmar AS, Rusli R, Hidayat R (2022). The application of K-means clustering for province clustering in Indonesia of the risk of the COVID-19 pandemic based on COVID-19 data. Qual Quant.

[100] Sari HL, Suranti D, Zulita LN (2017). Implementation of k-means clustering method for electronic learning model. J Phys Conf Ser.

[101] Ben-David S, Pál D, Simon HU, Bshouty NH, Gentile C (2007). Stability of k-means clustering. Learning Theory.

[102] Calinski T, Harabasz J (1974). A dendrite method for cluster analysis. Comm Stats Simulation Comp.

[103] Lord E, Willems M, Lapointe F, Makarenkov V (2017). Using the stability of objects to determine the number of clusters in datasets. Inform Sci.

